# Multienzymes activity of metals and metal oxide nanomaterials: applications from biotechnology to medicine and environmental engineering

**DOI:** 10.1186/s12951-021-00771-1

**Published:** 2021-01-19

**Authors:** Negar Alizadeh, Abdollah Salimi

**Affiliations:** 1 Department of Chemistry, University of Kurdistan, 66177-15175 Sanandaj, Iran; 2 Research Center for Nanotechnology, University of Kurdistan, 66177-15175 Sanandaj, Iran

**Keywords:** Nanozyme, Metal, Metal oxide, Sensing and biosensing, Cancer, Therapeutic, Diagnostics

## Abstract

With the rapid advancement and progress of nanotechnology, nanomaterials with enzyme-like catalytic activity have fascinated the remarkable attention of researchers, due to their low cost, high operational stability, adjustable catalytic activity, and ease of recycling and reuse. Nanozymes can catalyze the same reactions as performed by enzymes in nature. In contrast the intrinsic shortcomings of natural enzymes such as high manufacturing cost, low operational stability, production complexity, harsh catalytic conditions and difficulties of recycling, did not limit their wide applications. The broad interest in enzymatic nanomaterial relies on their outstanding properties such as stability, high activity, and rigidity to harsh environments, long-term storage and easy preparation, which make them a convenient substitute instead of the native enzyme. These abilities make the nanozymes suitable for multiple applications in sensing and imaging, tissue engineering, environmental protection, satisfactory tumor diagnostic and therapeutic, because of distinguished properties compared with other artificial enzymes such as high biocompatibility, low toxicity, size dependent catalytic activities, large surface area for further bioconjugation or modification and also smart response to external stimuli. This review summarizes and highlights latest progress in applications of metal and metal oxide nanomaterials with enzyme/multienzyme mimicking activities. We cover the applications of sensing, cancer therapy, water treatment and anti-bacterial efficacy. We also put forward the current challenges and prospects in this research area, hoping to extension of this emerging field. In addition to therapeutic potential of nanozymes for disease prevention, their practical effects in diagnostics, to monitor the presence of SARS-CoV-2 and related biomarkers for future pandemics will be predicted.
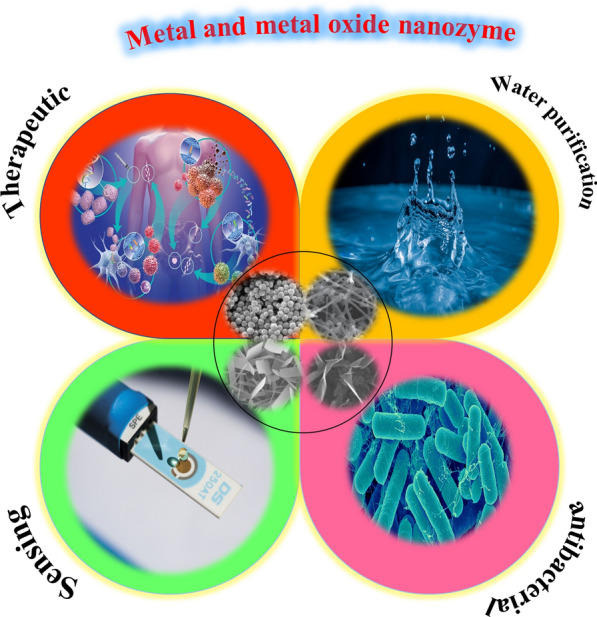

## Introduction

Enzymes, as biological macromolecules, are mainly composed of proteins, which can efficiently and selectively catalyze a diverse biochemical reactions [1, 2]. They play a notable function in various fields, such as energy production processes, biosensing, the food industry, and biofuels [3–6]. However, they have some drawbacks, such as product complexity, harsh catalytic conditions and low operational stability because of digestion and denaturation. In addition, it has high costs in preparation and purification [7, 8]. To address these issues, nanomaterial with enzyme-like characteristics (nanozyme) was applied as a novel alternative candidate. Artificial enzymes have attracted the significant attention of researchers due to their higher stability, low cost, flexibility and tunable catalytic activities [9–11]. Compared with other artificial enzymes, nanozymes possess outstanding properties such as their size and structure dependent catalytic activities, multi enzyme activity, large surface area, smart response and self-assembly capability [12, 13]. On the basis of these outstanding properties, nanozymes have been widely utilized for disease diagnosis and treatment, chemical sensing, environmental protection and antibacterial agents [7, 14–17]. Up to now, lots of nanomaterials have been uncovered to mimic several natural enzymes, such as peroxidase, oxidase, catalase, superoxide dismutase (SOD), phosphatase, nuclease, esterase, protease and ferroxidase [18]. Since the finding of Fe_3_O_4_ nanoparticles as peroxidase mimics in 2007 [19], a large amount of studies on metal and metal oxide nanozymes have been reported. For example, Au, Pt, Pd, Co_3_O_4_, CeO_2_, CuO, MnO_2_, NiO, V_2_O_5_ nanocomposites have been shown to possess a unique enzyme-like property [20–28]. Metal and metal oxide nanomaterial played great role in progress and development of enzyme mimic technology, due to their unique combination of redox chemistry, optical and electrical properties [29–32]. Interestingly, some nanomaterial can mimic the function of two or three enzymes. It has been reported that the simultaneous expression of multiple enzymes is more effective than single expression to remove harmful reactive oxygen species [33]. When designing a cascade reaction, it is often appropriate to use multiple nanozyme as the cascade catalyst. In this review, we present a comprehensive review of applications of metal and metal oxide nanozyme in terms of chemical sensing and biosensing, cancer treatment, water purification and anti-bacterial efficiency (Table 1). We also highlight some recent examples of multi-enzyme applications in catalysis. Because of the space limit, we could not cover all the related publications. However, we summarize recent research works on metal and metal oxide based nanozyme in Table 1. In the last section, the current challenges and future opportunities of metal and metal oxide-based nanozymes are also discussed. We hope that the present review will be of great benefit for development of novel nanozymes in the fields of medicine, chemistry, biology and nanotechnology.

**Table 1:** Current metal and metal oxide nanozymes, their typical applications and representative references

Enzyme	Nanomaterial	Application	References
Peroxidase	HccFn(Co_3_O_4_)	Sensing	[37]
Peroxidase	Co_2_(OH)_2_CO_3_-CeO_2_	Sensing	[43]
Peroxidase	Iron oxide	Sensing	[45]
Peroxidase	PtPd	Sensing	[49]
Oxidase	Ag-CoFe_2_O_4_/rGO	Sensing	[55]
Peroxidase	CuO/ Pt	Sensing	[56]
Peroxidase	(rGO)-PdAu	Sensing	[60]
Peroxidase	Au	Sensing	[61]
GSH-oxidase and peroxidase	CuO	Sensing	[65]
Peroxidase	Au@Pt	Sensing	[70]
Peroxidase	GO–AuNP	Sensing	[71]
Peroxidase	Cu_2_O/rGO	Sensing	[74]
Oxidase	CoOOH	Sensing	[77]
Peroxidase	FeMnO_3_	Sensing	[80]
Peroxidase	CuFe_2_O_4_/Cu_9_S_8_/PPy	Sensing	[81]
Peroxidase	CuO	Sensing	[85]
Peroxidase	Fe_3_O_4_ NPs/rGO/MoS_2_	Sensing	[86]
Peroxidase	CuO/WO_3_-GO	Sensing	[91]
Peroxidase	Pt–Pd	Sensing	[97]
GSH-oxidase and peroxidase	MnO_2_	Therapeutics	[110]
Peroxidase	FcPW	Therapeutics	[114]
Peroxidase	Copper peroxide	Therapeutics	[116]
Peroxidase	SnFe_2_O_4_	Therapeutics	[118]
Peroxidase	Fe_3_O_4_@MSN	Therapeutics	[122]
Catalase	MnFe_2_O_4_	Therapeutics	[130]
Catalase	MnO_2_	Therapeutics	[132, 133]
Catalase	Pt	Therapeutics	[134]
Catalase and oxidase	MoO_3_ − x	Therapeutics	[136]
Peroxidase and oxidase	Au@HCNs	Therapeutics	[144]
Catalase and superoxide dismutase	NCeO_2_-PEI-MoS_2_	Therapeutics	[145]
Catalase	Pt-CuS	Therapeutics	[151]
Peroxidase and oxidase	GQD/AgNP	Antibacterial	[159]
Oxidase, peroxidase and catalase	Pt/Ag	Antibacterial	[160]
Oxidase and peroxidase	MSN-Au	Antibacterial	[164]
Peroxidase	CuO	Antibacterial	[167]
Oxidase and peroxidase	Pd	Antibacterial	[170]
Peroxidase	Fe_3_O_4_	Water purification	[182, 185, 188]
Peroxidase	Fe_2_O_3_	Water purification	[183]
Peroxidase	CuFe_2_O_4_	Water purification	[184]
Peroxidase	Fe_2.79_Nb_0.19_O_4_	Water purification	[187]

## Nanozymes for sensing application

Metal and metal oxide-based nanozymes with substantial properties have been widely applied for several analytical purposes. The principle detection is divided into two categories: (1) the target activates or deactivates a reaction between the nanozyme and the agent, (2) the presence of the nanozyme and its reaction with the agent indirectly indicates the amount of target. According to previous reports, the application of such nanozymes includes detection of a variety of important targets, such as tumor markers, small biomolecules and metal ions [18, 28, 34].

## Tumor markers

**Fig. 1:**
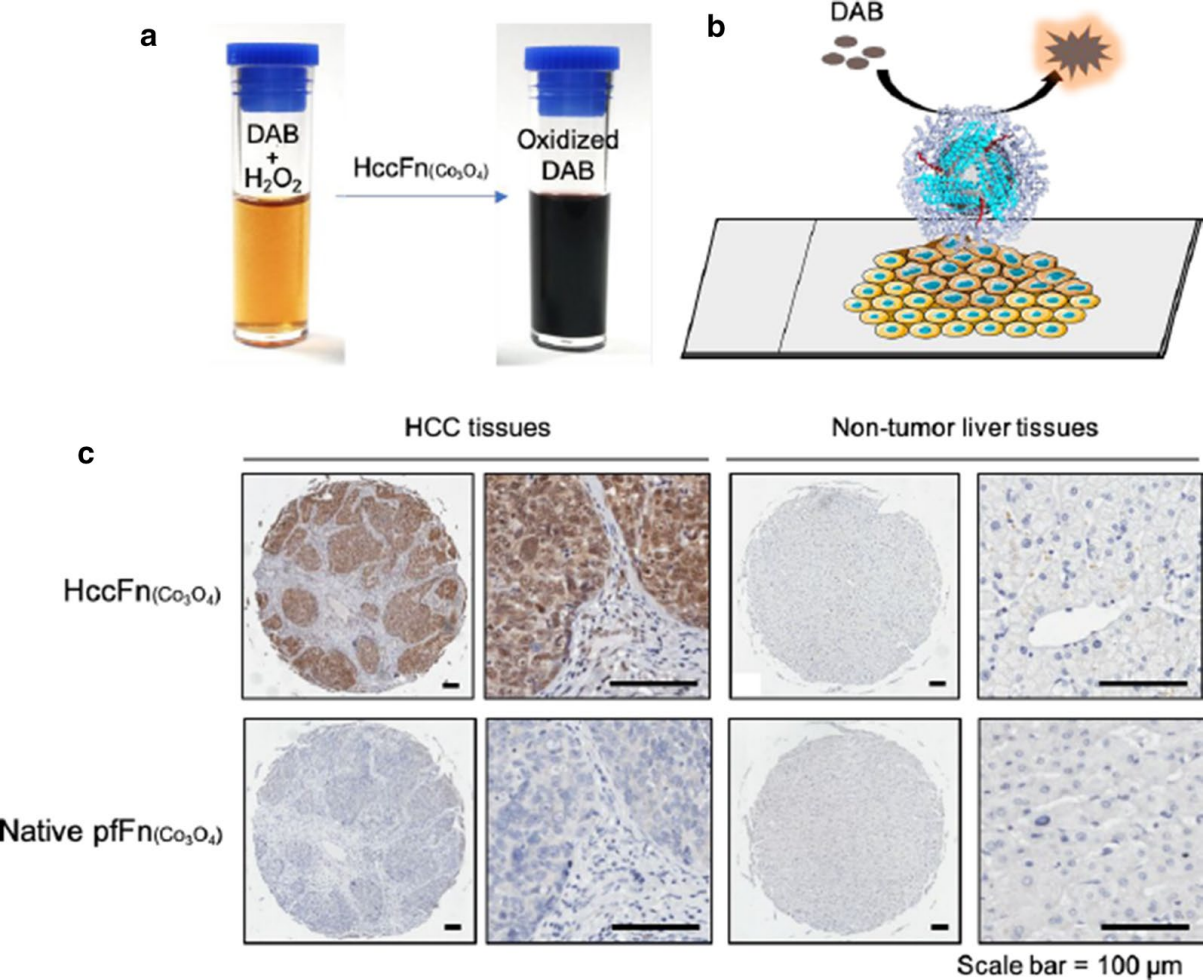
HccFn(Co_3_O_4_) nanozymes specifically recognize and visualize clinical HCC tissues. **a** HccFn(Co_3_O_4_) nanozymes showed peroxidase-like activity and catalyzed the oxidation of peroxidase substrate diaminobenzidine (DAB) to produce colorimetric reaction. **b** Schematic diagram of HccFn(Co_3_O_4_)-based immunohistochemical approach. **c** HccFn(Co_3_O_4_)-based immunohistochemical staining (top row) and Fn(Co_3_O_4_)-based immunohistochemical staining (bottom row) of HCC tissues and non-tumor liver tissues

Synthesize nanomaterials within cage-like protein templates has been demonstrated to be a suitable approach to produce uniform [35]. Ferritin nanocages provide surface modification and specific targeting abilities for synthesizing ferritin-based nanozymes [36]. Biomineralization synthesis of cobalt nanozyme in SP94-ferritin nanocage was reported for prognostic diagnosis of hepatocellular carcinoma (HCC) [37]. In this report, ferritin-based cobalt nanozyme (HccFn(Co_3_O_4_)) was designed for HCC diagnosis and therapy. SP94 peptide was modified onto the exterior surface of ferritin nanocage (HccFn) for specifically binding to HCC cells. HccFn(Co_3_O_4_) nanozymes specifically bound to HCC tissues and catalyze the oxidation of peroxidase substrate diaminobenzidine (DAB) to produce deep brown colorimetric reaction. In comparison with Fn(Co_3_O_4_) control group, HccFn(Co_3_O_4_) nanozymes specifically recognized and visualized HCC tissues and could distinguish tumor cells from normal tissues (Fig. 1).

**Fig. 2:**
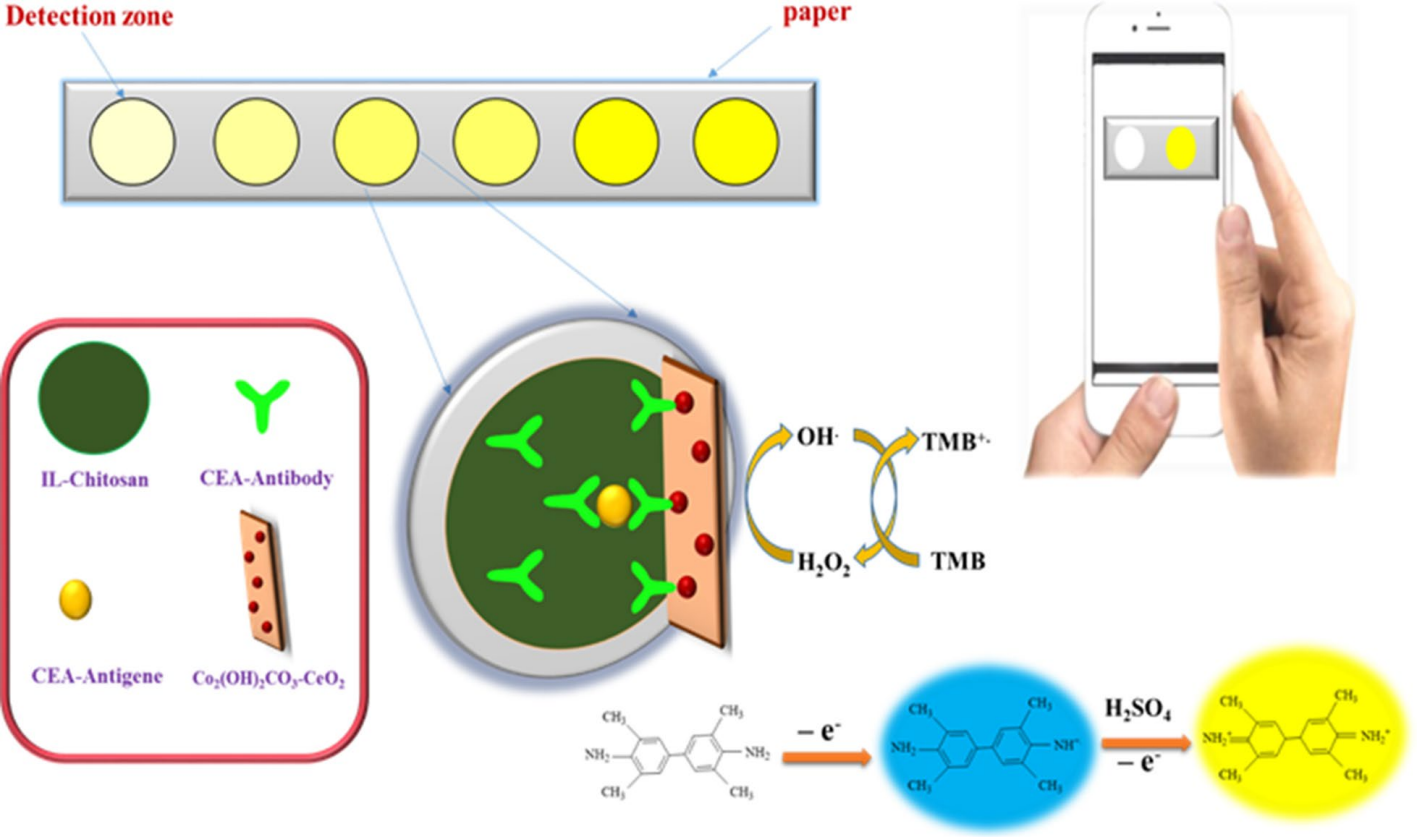
Schematic illustration and assay procedure of CEA detection on the paper-based chip

The nanomaterial-mediated colorimetric sensor is an attractive system for advance instrument-free bioanalysis due to its unique advantages of simplicity in operating analysis via camera or smartphone [38, 39]. Several colorimetric assays based on the 3,3,5,5 tetramethylbenzidine (TMB)-H_2_O_2_ system catalyzed by enzyme mimic nanomaterials have been extensively developed for immunoassay [40–42]. For instance, Alizadeh et al. present a paper-based microfluidic colorimetric immunosensor for the detection of carcinoembryonic antigen (CEA), using Co_2_(OH)_2_CO_3_-CeO_2_ nanocomposite with extraordinary intrinsic peroxidase like activity [43]. The proposed immunosensor facilely prepared by modifying mixture of ionic liquid and chitosan functionalized primary antibodies (Ab_1_) on the surface of paper. Co_2_(OH)_2_CO_3_-CeO_2_ peroxidase mimicking enzyme was functionalized secondary antibodies (Ab_2_) and used as a signal tag. Co_2_(OH)_2_CO_3_–CeO_2_ nanocomposite catalyzed the oxidation of 3,3′,5,5′-tetramethyl benzidine in the presence of H_2_O_2_, resulting in a color change, which acquired as the immunosensor response. The color change was distinct by the naked eye and analyzed by an installed application on the smartphone (Fig. 2).

**Fig. 3:**
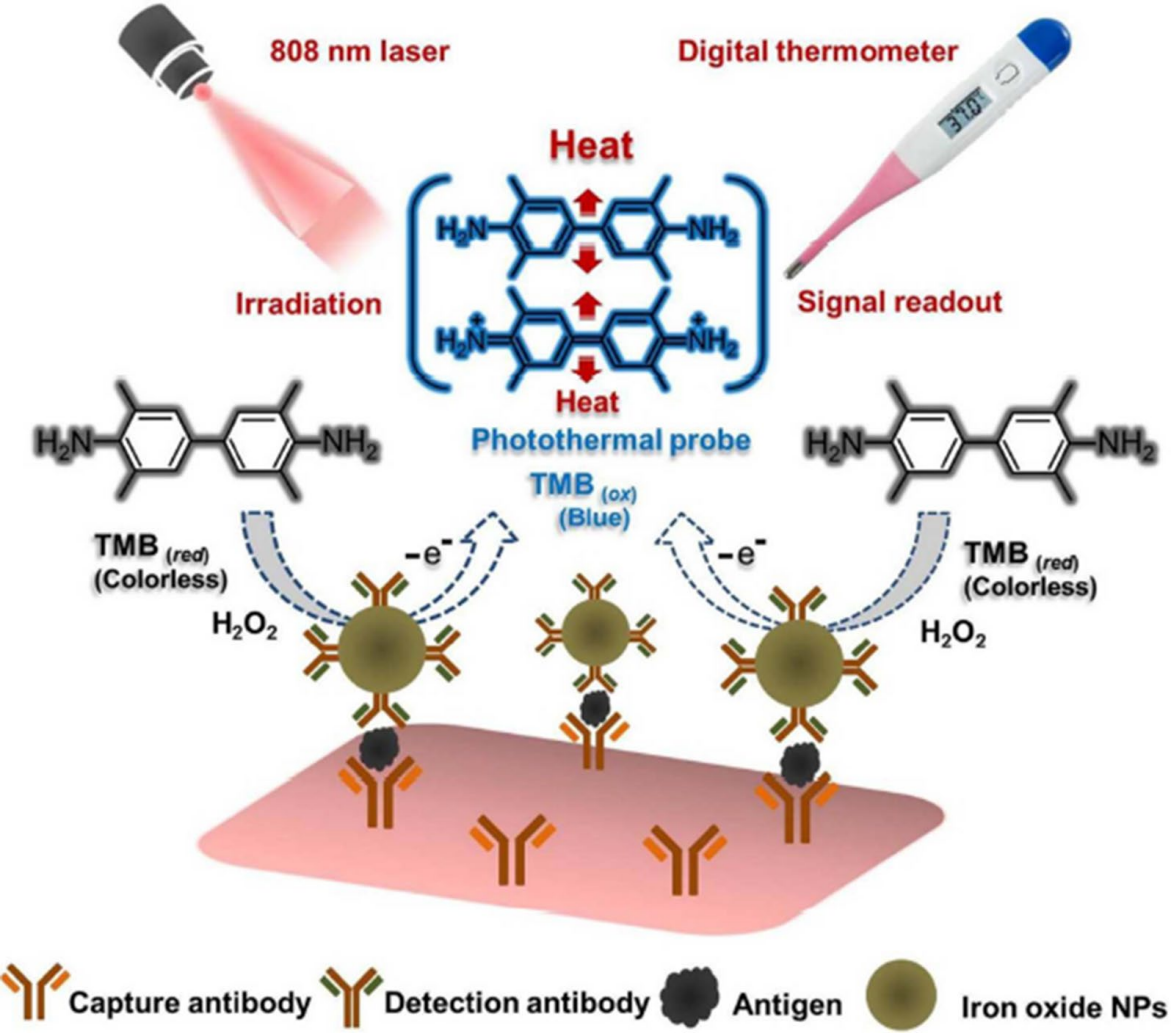
Schematic illustration of the photothermal immunoassay platform based on the photothermal effect of the iron oxide NPs mediated TMB-H_2_O_2_ colorimetric system

In colorimetric assays, color changes and photothermal effect of TMB-H_2_O_2_ colorimetric system have been prospected [44]. In this regard, nanoparticle (NPs)-mediated photothermal immunoassay platform was developed for detection of prostate-specific antigen (PSA) using a common thermometer as the quantitative signal reader [45]. The iron oxide NPs-labeled antibody was applied as the detection probe, on basis of sandwich-type proof-of-concept immunoassay. In the immunoassay, iron oxide artificial enzyme demonstrated color changes and also a strong NIR laser-driven photothermal effect, simultaneously. The oxidized TMB acted as a highly sensitive photothermal probe to convert the immunoassay signal into heat via its photothermal effect (Fig. 3).

**Fig. 4:**
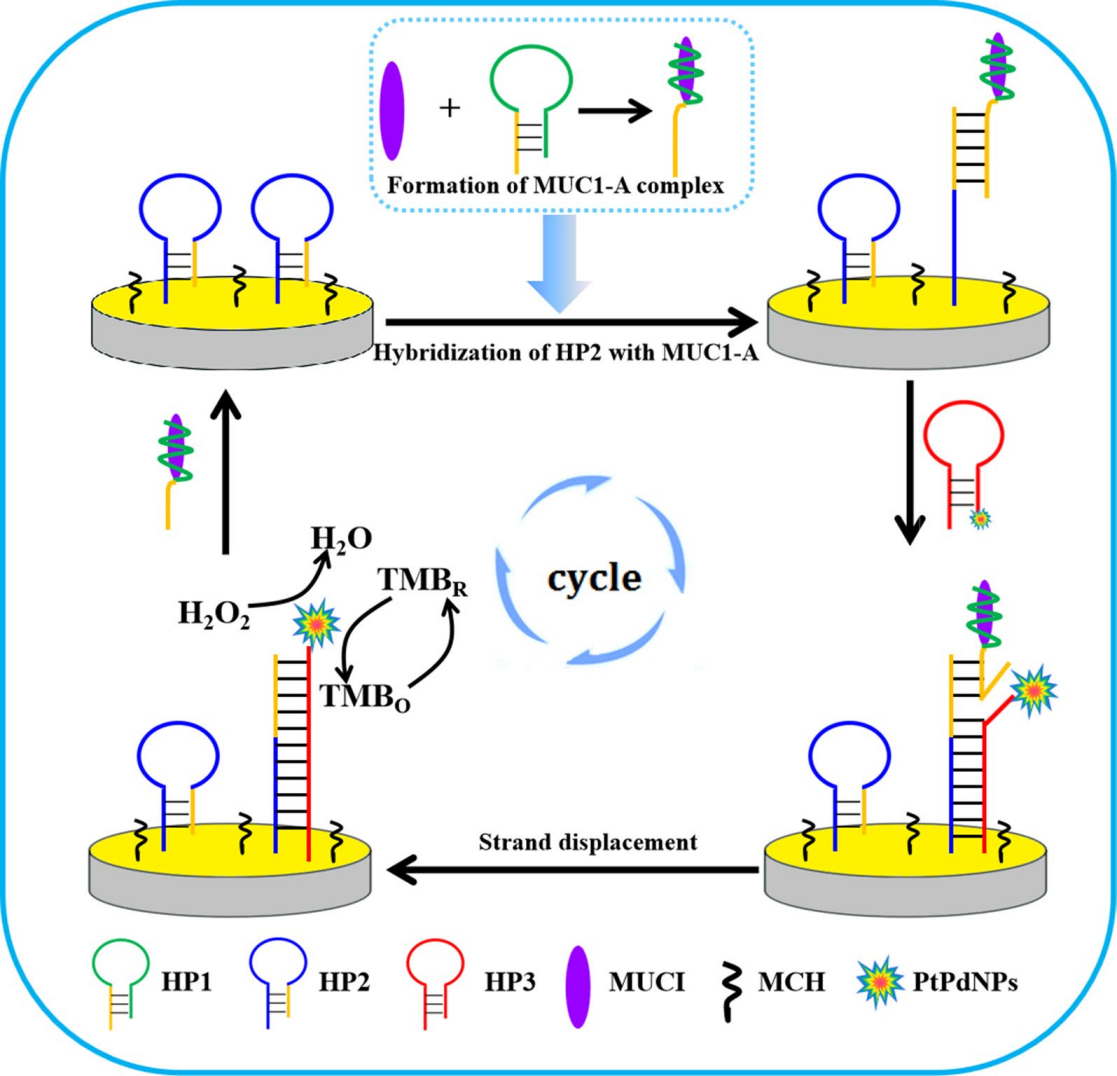
Schematic representation of the aptasensor for the detection of MUC1

Aptamers are artificial synthetic single-stranded DNA or RNA oligonucleotides, which can bind with various targets such as protein, peptide, organic/inorganic molecule, and cell with high affinity and specificity. Aptamer with superiority to antibodies, including high stability, ease of synthesis, low cost and easy chemical modification, have attracted a lot of attention in biomedical and bioanalysis research [46–48]. Zhao et al. selected three hairpin anti-MUC1 DNA aptamers for construction of a sensitive electrochemical aptasensor based on catalytic hairpin assembly coupled with PtPdNPs peroxidase-like activity [49]. After binding with target protein, Apt-HP1 containing aptamer sequence was opened and MUC1-aptamer binding complex formed (Fig. 4). Next, the exposed segment of HP1 would attack HP2 immobilized on the electrode to form a double strand structure. Then, the new exposed segment of HP2 hybridized with the toehold of PtPdNPs modified HP3. Finally, MUC1-A was released via the strand displacement process, and the released MUC1-A could participate in the subsequent reaction cycles. The carried PtPdNPs, as a mimic peroxidase probe, catalyzed the TMB by H_2_O_2_, leading to the electrochemical signal generation.

## Metal ions

Most studies on metal ions sensing with nanozymes have been devoted to mercury ions (Hg^2+^) [9, 50, 51]. Mercury is a toxic metal ion that in the environment can produce several harmful effects on people`s health like brain, heart, kidneys and central nervous system damages [52, 53]. Through the bacteria action in a lake and ocean, Hg^2+^ converts into more toxic organic mercury and accumulates in aquatic organisms [54]. Thereupon, it can accumulate continuously in the body through water and food. In a report, dual colorimetric and SERS detection of Hg^2+^ was developed based on the stimulus of intrinsic oxidase-like catalytic activity of Ag-CoFe_2_O_4_/rGO nanocomposites [55]. CoFe_2_O_4_ nanoparticles in Ag-CoFe_2_O_4_/rGO nanocomposites exhibited an oxidase-like activity, which can quickly catalyze the oxidation of typical chromogenic substrates 3,3ʹ,5,5ʹ-tetramethylbenzidine (TMB) in the presence of dissolved oxygen. The introduction of Hg^2+^ led to enhancement in oxidase-like activity of the Ag-CoFe_2_O_4_/rGO nanocomposites due to the formation of the Ag-Hg alloy. Owing to the existence of the Ag nanoparticles the prepared nanocomposites have also been demonstrated to be efficient SERS substrate. In another report, colorimetric detection of Hg^2+^ in various groundwater samples was successfully performed using CuO/ Pt Nanoflowers (NFs). In the presence of Hg^2+^, the peroxidase activity of CuO/Pt NFs was hindered, because the formation of the CuO/Pt-Hg trimetallic amalgam [56].

**Fig. 5:**
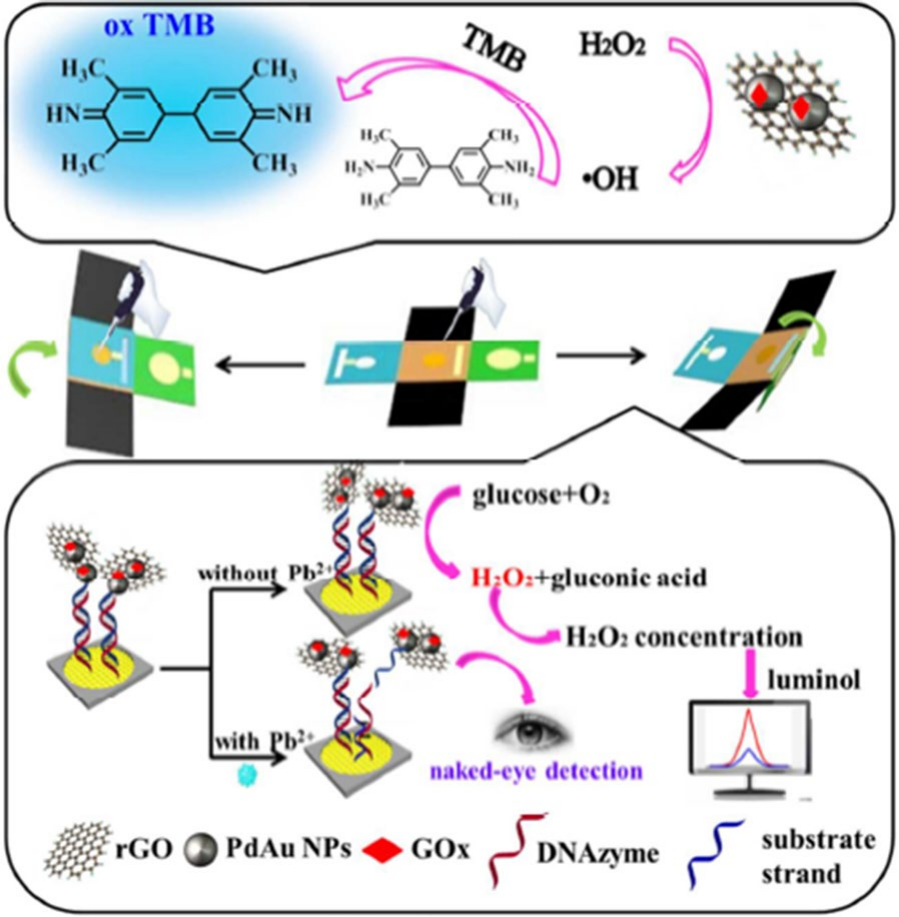
Schematic representation of fabrication procedures of the lab-on-paper device and dual mode sensing mechanism

Lead (Pb^2+^) is known as a non-biodegradable, toxic and perdurable metal ion. It has a strong negative effect on children’s behavior and serious damage to the brain, immune system of many life tissues including liver, brain, kidney and also immune and central nervous system [57–59]. Xu et al. reported colorimetric and electrochemiluminescence dual mode sensing of lead ion biomolecules using graphene oxide (rGO)-PdAu probe [60]. Pb^2+^-specific DNAzyme was immobilized onto rGO-PdAu-glucose oxidase (GOx). The thiol modified Pb^2+^-dependent DNAzyme was self-assembled onto the surface of the flower-like Au NPs modified ECL detection zone to hybridize with rGO-PdAu-GOx labeled oligonucleotide. Upon introducing of Pb^2+^ into the prepared system, the double helix structure of DNA was cleaved, resulting in the release of rGO-PdAu-GOx probe to catalyze the oxidation and color change of TMB. Meanwhile, the concentration of H_2_O_2_ is proportional to the luminol ECL system, which constitutes a new mechanism for ECL detection of Pb^2+^ (Fig. 5).

Inspired by the aggregation-induced emission (AIE) properties, the catalytic activity of metal nanozyme could be altered upon aggregation, because both optical and catalytic properties of metal nanocomposites are highly dependent on their size and morphology. It was found that the Pb^2+^-induced aggregation can greatly accelerate the peroxidase-like activity of Au nanoclusters (Au-NCs) [61]. In the absence of Pb^2+^, Au-NCs could catalyze the TMB oxidation by H_2_O_2_ in a relatively slow reaction rate. After the Pb^2+^-induced aggregation, the peroxidase activities of Au-NCs toward oxidation of TMB substrate in the presence of H_2_O_2_ are nearly tenfold increased.

Some cascade reactions were configured between enzyme-like nanomaterials and natural enzymes [62, 63]. This cascade catalytic system must be carried out in two steps, because the optimal pH conditions for each enzyme is different. To address the limitation of different conditions, a single nanomaterial with dual activity has been constructed to mimic enzyme cascade reaction [64]. He et al. described a self-cascade system based on cupric oxide nanoparticles as dual-functional enzyme mimics for ultrasensitive detection of silver ions [65]. Cupric oxide nanoparticles (CuO NPs), as the dual-functional nanozyme, demonstrated the intrinsic GSH-oxidase and peroxidase-like activity coupling with terephthalic acid (TA) and GSH to construct a self-cascade fluorescent system. CuO NPs effectively catalyze the oxidation of GSH by oxygen to produce glutathiol (GSSG) and hydrogen peroxide, following to catalyze the decomposition of hydrogen peroxide into hydroxyl. Then, a highly fluorescent product TAOH was formed by oxidation of terephthalic acid (TA) in the presence of hydroxyl radical. Thus, in the presence of GSH, the turn-on fluorescence signal of oxidation hydroxyterephthalate (TAOH) is created. Introduction of the Ag^+^ ions cause to inhibition of the fluorescence of H_2_O_2_-TA-CuO NPs reaction system. It is due to that Ag^+^ ions can react with the H_2_O_2_ intermediate product resulted from the oxidation of GSH.

Until now, many assays for single heavy metal ions have been reported [66–68]. However, some efforts have been made to develop simultaneous detection of metal ions. Hg^2+^ and Ag^+^ are usually coexisting in water, soil and even biological systems [69]. Peng et al. prepared core–shell Au@Pt nanoparticles for simultaneous colorimetric detection of Hg^2+^ and Ag^+^ [70]. Both Hg^2+^ and Ag^+^ were found to intensively inhibit the catalytic activity of Au@Pt NPs. The complexation of sodium dodecyl sulfate (SDS) shields interference metal ions such as Mn^2+^, Sr^2+^, Zn^2+^, Fe^3+^, Co^2+^, Cu^2+^ and Bi^3+^, to obtain specific respond of Hg^2+^ and Ag^+^. As well as, L-cysteine can be used to mask Hg^2+^ in the presence of Ag^+^. In another study, Colorimetric detection of Hg^2+^ and Pb^2+^ was achieved based on peroxidase-like activity of graphene oxide–gold (GO–AuNP) nanohybrids [71]. Single-stranded DNA (ssDNA) were stable against the salt-induced aggregation of GO–AuNP nanohybrids, whereas double stranded DNA (dsDNA) did not hinder salt-induced GO–AuNP nanohybrids aggregation. On the basis of the ability of GO–AuNP nanohybrids to differentiate between ssDNA and dsDNA, label-free colorimetric method for the detection of Hg^2+^ and Pb^2+^ was developed. With addition of Hg^2+^ or Pb^2+^, ssDNA formed a hairpin-like or a quadruplex structure, and these conformational changes led to the salt-induced aggregation of GO AuNP nanohybrids. After addition of TMB and H_2_O_2_, the colorimetric signal was significantly decreased compared to that in the absence of Hg^2+^ or Pb^2+^.

## Biomolecules

**Fig. 6:**
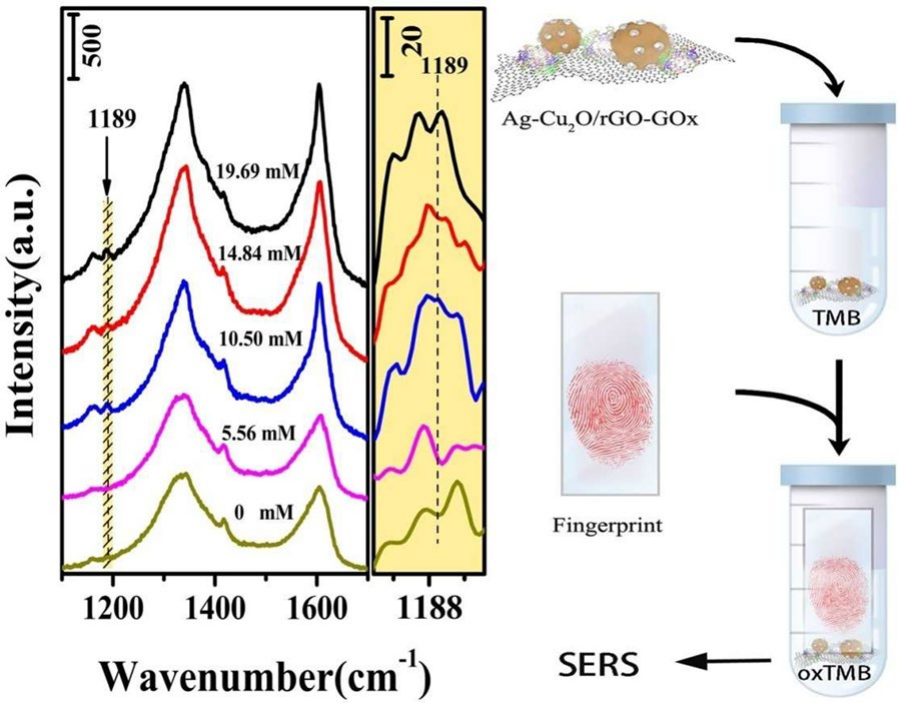
SERS spectra of oxidized TMB molecules on the surface of fingerprint from diabetes patients and normal pers in the presence of Ag-Cu_2_O/rGO substrates and GOx. The bottom line is blank test without fingerprint. On the right is schematic representation of SERS detection of fingerprint by using Ag-Cu_2_O/rGO nanocomposites as SERS substrate

Glucose is the main energy source for cellular metabolism and function of human bodies. However, people with glucose excessive suffer from diabetes mellitus. Diabetes can cause serious health problems, such as strokes, heart attacks, high blood pressure, and even blindness or death [72, 73]. Guo and co-workers fabricated Ag-Cu_2_O/reduced graphene oxide nanocomposites with peroxidase-like catalytic reaction for (Surface-enhanced Raman spectroscopy) SERS detection of glucose [74]. SERS facilitate highly sensitive and selective identification of analytes such as glucose. Ag-Cu_2_O/rGO nanocomposites operate as both peroxidase nanozyme and SERS substrates; they speed up the reaction between TMB and H_2_O_2_. A SERS method has been designed based on the ability of glucose oxidase (GOx) to catalyze the oxidation of glucose to gluconolactone and H_2_O_2_. In this method, glucose was determined by the catalytic oxidation of TMB in the presence of GOx and glucose. Discern between diabetic and normal individuals by determining the glucose levels within a fingerprint is the most important feature of this work (Fig. 6).

**Fig. 7:**
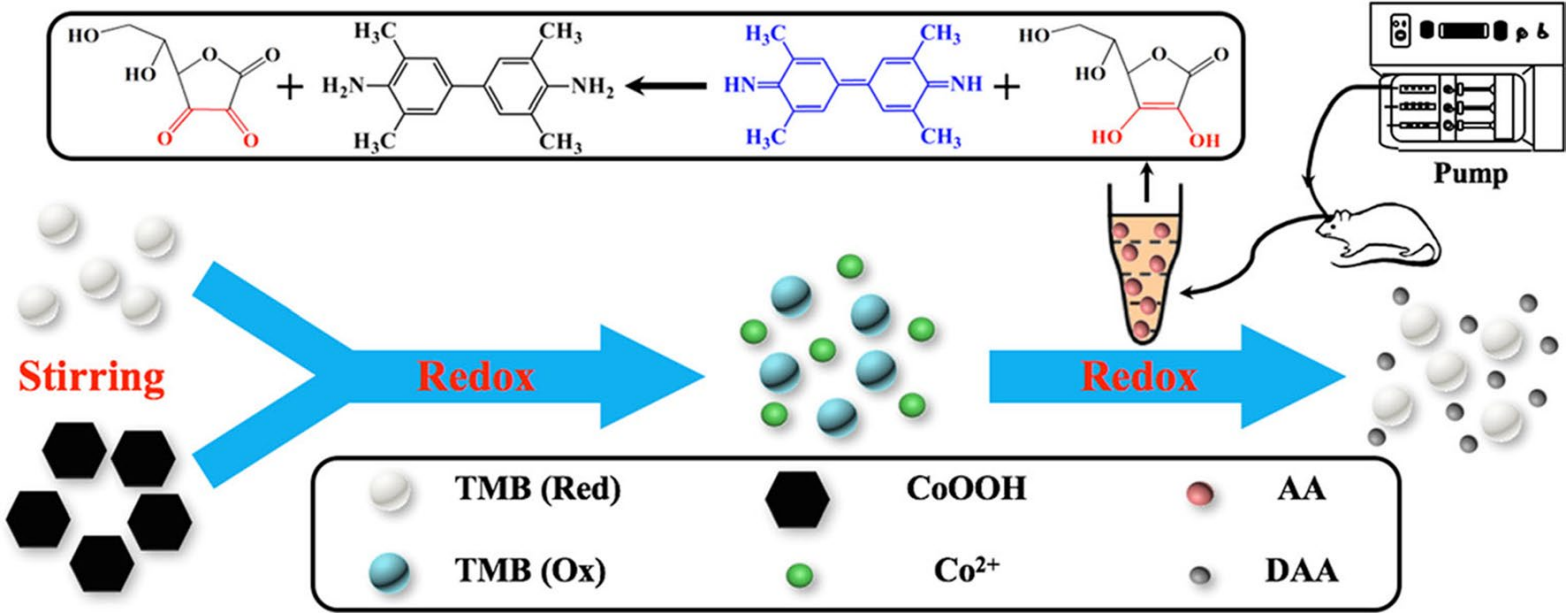
Schematic of colorimetric assay mechanism and platform for the detection of AA in rat brain by the CoOOHTMB system

Ascorbic acid (AA) neurochemicals, used as an enzyme cofactor and antioxidant. Meanwhile, AA plays a critical role of anti-oxygenation and resists the cells damage from free radicals. High level of AA can selectively kill colorectal cancer cells as a pro-oxidant anticancer agent [75, 76]. Therefore, methods for simple, fast, and effective AA assay suitable for the biological systems are required. Ding et al. the CoOOH-TMB oxidative system for colorimetric and test strip based detection of ascorbic acid [77]. CoOOH nanoflakes directly oxidize TMB (colorless) to blue oxTMB with a characteristic absorption peak at 652 nm. In the presence of ascorbic acid (AA), the absorbance decreased because AA reduces oxTMB. Furthermore, the CoOOH-TMB systems can be further developed into a paper-strip-based assay for determination of AA in rat brain (Fig. 7).

By reacting with H_2_O_2_, some small bioactive molecules like dopamine and glutathione (GSH) have been determined based on their inhibition effects on peroxidase enzyme mimics [78, 79]. For example, GSH in human serum samples was determined using FeMnO_3_ nanoparticles-filled polypyrrole nanotubes as peroxidase mimic [80]. More, with Yang's sensing strategy, selective colorimetric detection of dopamine was successfully executed in real samples [81].

## Cancer cell and bacteria

**Fig. 8:**
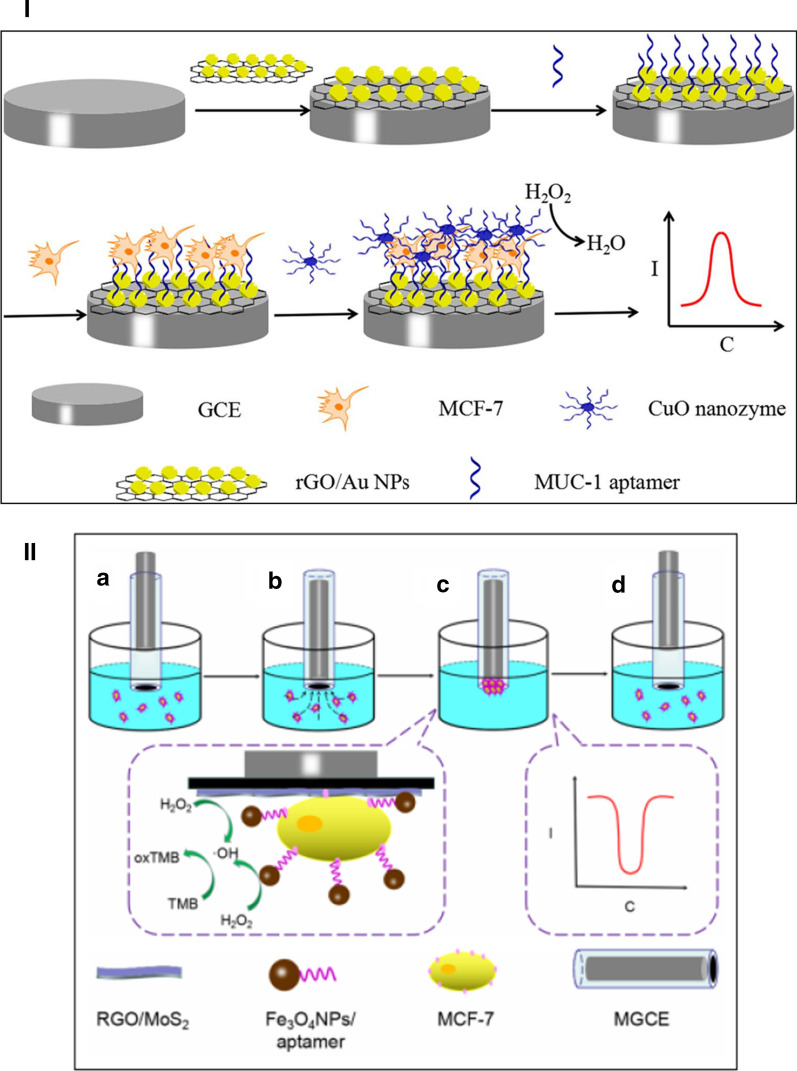
Schematic representation of the cytosensor for detection of MCF-7 using (I) CuO and (II) Fe_3_O_4_ nanozyme

Cancer is one of the fatal sicknesses and has become a major public worry in the world [82]. Presently, early diagnosis has been made to be the most effective way to raise survival rate [83]. Thus, it is highly needed to develop sensitive, rapid and specific methods to detect and quantification of cancer cells at early stage [84]. The conjugation of aptamer or a ligand with nanozymes can be employed for cancer cells detection. For instance, MCF-7 circulating tumor cells were detected by an electrochemical cytosensor with effective surface recognition between specific mucin 1 protein (MUC-1) over-expressed on the MCF-7 cell membranes and MUC-1 aptamer [85]. The CuO nanozyme was used as a signal-amplifying nanoprobe with reduced graphene oxide/gold nanoparticles composites (rGO/AuNPs composites) as a support material (Fig. 8I). The fabricated “sandwich” structure can help to reach on the acceptable sensitivity of the proposed cytosensor.

The immunomagnetic sensor was also developed for electrochemical detection of MCF-7 circulating tumor cells [86]. Fe_3_O_4_ NPs magnetic beads act as both separation and enrichment CTCs and as enzyme mimics with rGO/MoS_2_ synergistic catalysis (Fig. 8II). The CTCs could be separated and enriched on the magnetic glassy carbon electrode (MGCE) by Fe_3_O_4_ NPs coated aptamer. Electrochemical current of TMB redox product was generated via Fe_3_O_4_ NPs/rGO/MoS_2_ catalytic ability.

**Fig. 9:**
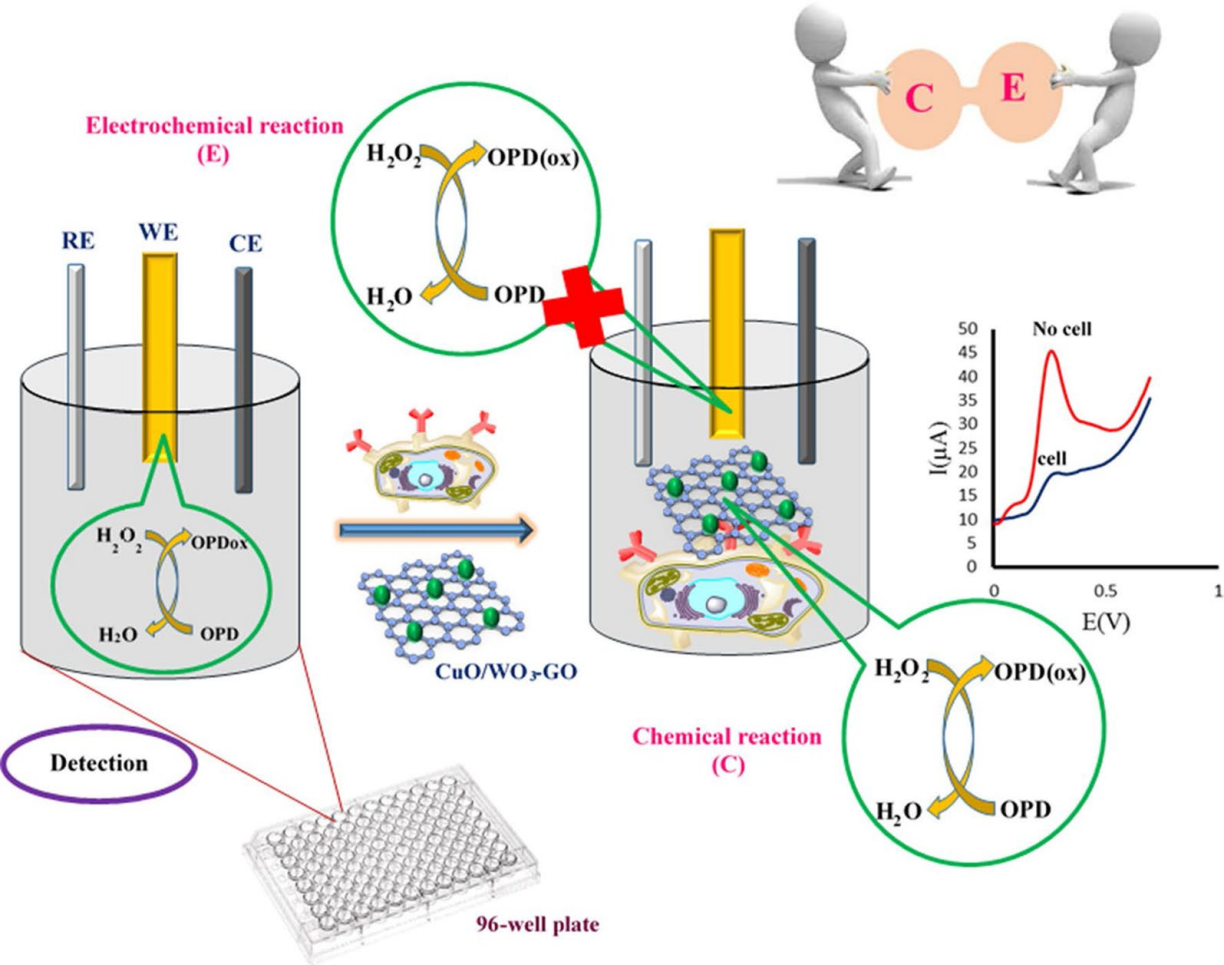
Schematic illustration of obtained chemical /electrochemical reactions and peroxidase activity of CuO/WO_3_-GO for cancer cell detection

Recently, several studies reported that folate-modified nanozymes could detect cancer cells with over-expressed folate receptor [87–90]. In Alizadeh and co-workers' study, the novel method was developed for electrochemical cancer cell detection using CuO/WO_3_ nanoparticle decorated graphene oxide nanosheet (CuO/WO_3_-GO) conjugated with folic acid (FA) [91]. In the absence of cancer cells, *o*-Phenylenediamine (OPD) oxidized on the Au electrode in the presence of H_2_O_2_, while FA/CuO/WO_3_-GO with peroxidase like activity reacted with folate receptor of cancer cells seeded on 96-well plate, catalyzed the oxidation of OPD in presence of H_2_O_2_ (Fig. 9). Actually, in the presence of cancer cells, the response signal decreased, because some amount of H_2_O_2_–OPD system participated in chemical reaction and removed from the electrode. In this way, cancer cells detected in wide linear range and a low detection limit.

Infectious diseases induced by bacteria considered a cause of more than 25% of all global deaths [92]. Pathogen detection is an important step in the inhibition of these types of infectious and deadly diseases [93]. Several studies have been employed for bacteria detection using nanozymes as probing elements [94–96]. Recently, Cheng et al. reported nanozyme mediated dual-immunoassay integrated with smartphone for use in simultaneous detection of pathogens [97]. They applied platinum-palladium (Pt–Pd) nanoparticles as a signal amplifier in a dual-lateral flow immunoassays (LFIA) and for simultaneous colorimetric detection of *Salmonella* Enteritidis and *E. coli* O157:H7. Smartphone-based device and its ability to image provide a portable and cost-effective platform for tracking bacterial contamination along the entire food chain.

## Nanozymes for therapeutics application

Reactive oxygen species (ROS), is a general expression that describes the chemical species generated upon incomplete reduction of oxygen [98]. ROS including hydrogen peroxide, superoxide anion, hydroxyl radical and singlet oxygen have the potential to kill cancer cells by destroying biomolecules such as DNA, proteins and lipids [99]. In recent years, substantial achievements have been made in ROS-based nanomedicine, especially in cancer and bacterial infection [100–102]. The development of nanotechnology has favored the production of several ROS-generation materials with enzyme-mimic characteristics [103]. Metal and metal oxide nanozymes with peroxidase- and catalase- like activities, can convert endogenous biological H_2_O_2_ into highly cytotoxic OH^·^ and O_2_^·–^ species [104]. Furthermore, developing nanotechnology and nanozymes with special ROS-regulating properties solve the problem of the instability of ROS-based therapeutics. In this section, nanozymes catalytic mediated cancer therapies are discussed, which cover (photodynamic therapy) PDT, chemodynamic therapy (CDT), sonodynamic therapy (SDT) and photothermal therapy (PTT). Furthermore, the Metal and metal oxide-based nanozymes for antimicrobial therapies are summarized.

## Chemodynamic therapy (CDT)

**Fig. 10:**
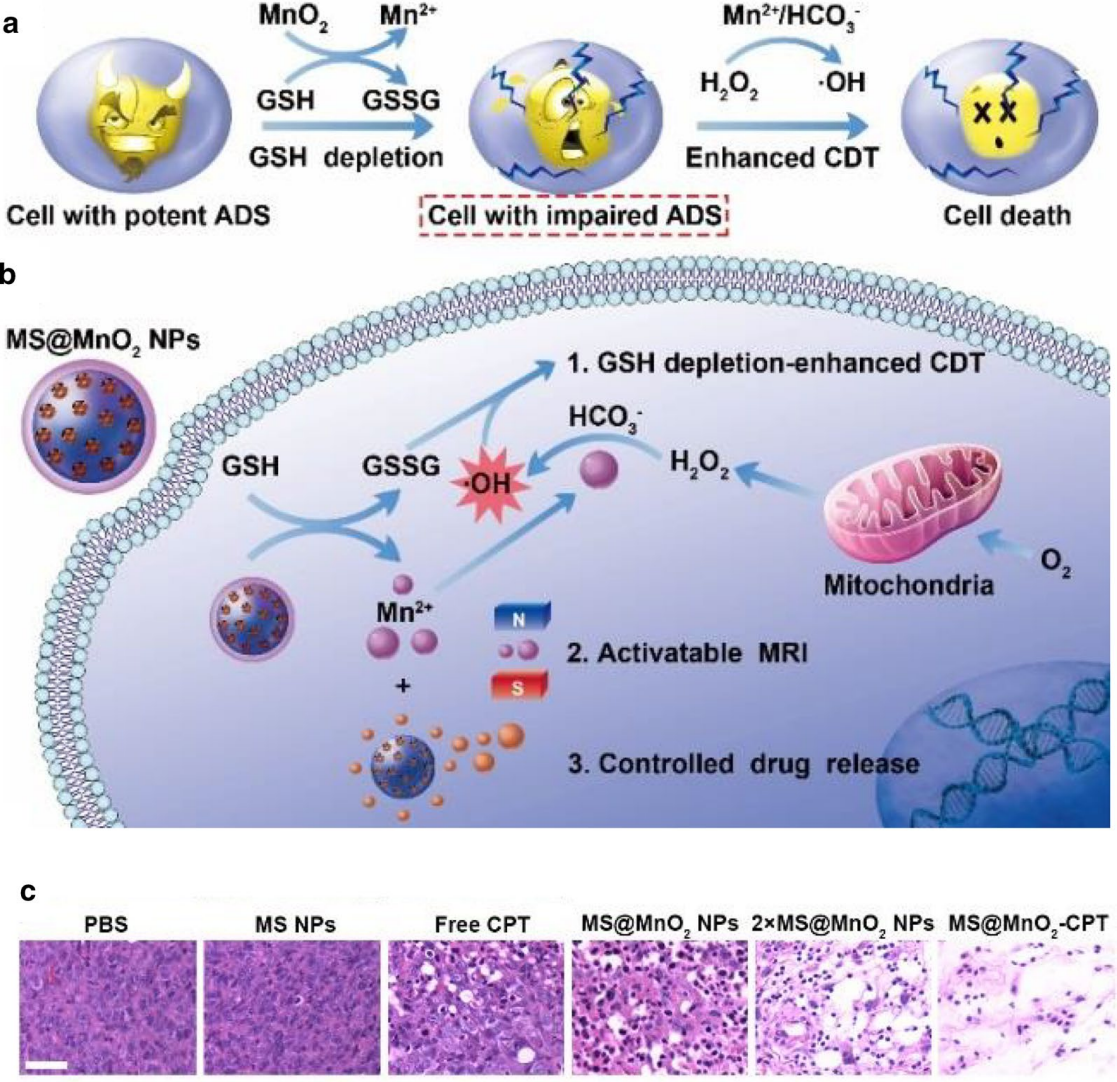
**a** The mechanism of MnO_2_ as a smart chemodynamic agent for enhanced CDT of cancer. Upon endocytosis, the MnO_2_ can react with intracellular GSH to produce GSSG and Mn^2+^, which exerts excellent Fenton-like activity to generate highly reactive OH· from endogenous H_2_O_2_ in the presence of physiological HCO3-. The impairment of antioxidant defense system (ADS) resulting from GSH depletion makes cancer cells more vulnerable to OH· formed in Mn^2+^-mediated Fenton-like process, enabling enhanced CDT. **b** Schematic illustrating the application of MS@MnO_2_ NPs for MRI-monitored chemo-chemodynamic combination therapy. **c** H&E-stained images of tumor sections from different groups. Scale bar, 100 μm

Chemodynamic therapy (CDT) is an emerging cancer treatment strategy that damage tumor cells with a localized Fenton reaction. In CDT process iron mediated Fenton reaction induces intracellular oxidative stress by converting less reactive H_2_O_2_ into OH·, one of the most detrimental ROS [105, 106]. Up to now, iron oxide and other metal oxide nanocomposite enzyme mimic are capable of decomposing H_2_O_2_ into OH· through Fenton-like reactions [107]. Researchers have been widely studied CDT treatment method because of its high tumor specificity, lower side effects and minimal invasiveness. Generally, transition metal ions (e.g., Fe, Co, Ni, Cu, and Mn) used as the CDT agents to catalyze the decomposition of hydrogen peroxide (H_2_O_2_) and produce high-toxicity hydroxyl radicals (·OH) (Typical reaction: Fe^2+^  + H_2_O_2_ → Fe^3+^  + ·OH + OH^−^). Biomolecular substances including nucleic acids, lipids and proteins in tumor cells are destroyed as a result of oxidative stress [108, 109]. Conceivably, some challenges like overexpressed glutathione (GSH) and nicotinamide adenine dinucleotide phosphate (NADPH) in tumor cells, low H_2_O_2_ concentration and requirement of strong acidic chemical environment obstacles CDT. Lin et al. reported enhanced chemodynamic therapy based on MnO_2_ nanoagent with Fenton-like Mn^2+^ delivery and GSH depletion properties [110]. The MnO_2_ was established on the surface of thiol-functionalized mesoporous silica (MS) NPs, leading to the formation of MnO_2_-coated MS NPs (MS@MnO_2_ NPs). Upon uptake of the MS@MnO_2_ NPs by cancer cells, the MnO_2_ layer would simultaneously release Mn^2+^ with superior Fenton-like activity to transform endogenous H_2_O_2_ produced into the highly toxic OH· and deplete intracellular GSH to inhibit OH· scavenging (Fig. 10a, b). As well as, the potential of MnO_2_ shell as a gatekeeper for controlled drug release showed by loading hydrophobic anticancer drug camptothecin (CPT) into the PEGylated MS@MnO_2_ NPs. Hematoxylin and eosin (H&E)-stained images demonstrated that tumor tissues treated with MS@MnO_2_-CPT suffered more intense damage than other control group, which indicates enhanced chemodynamic efficacy of MS@MnO_2_-CPT for theranostic applications (Fig. 10c).

**Fig. 11:**
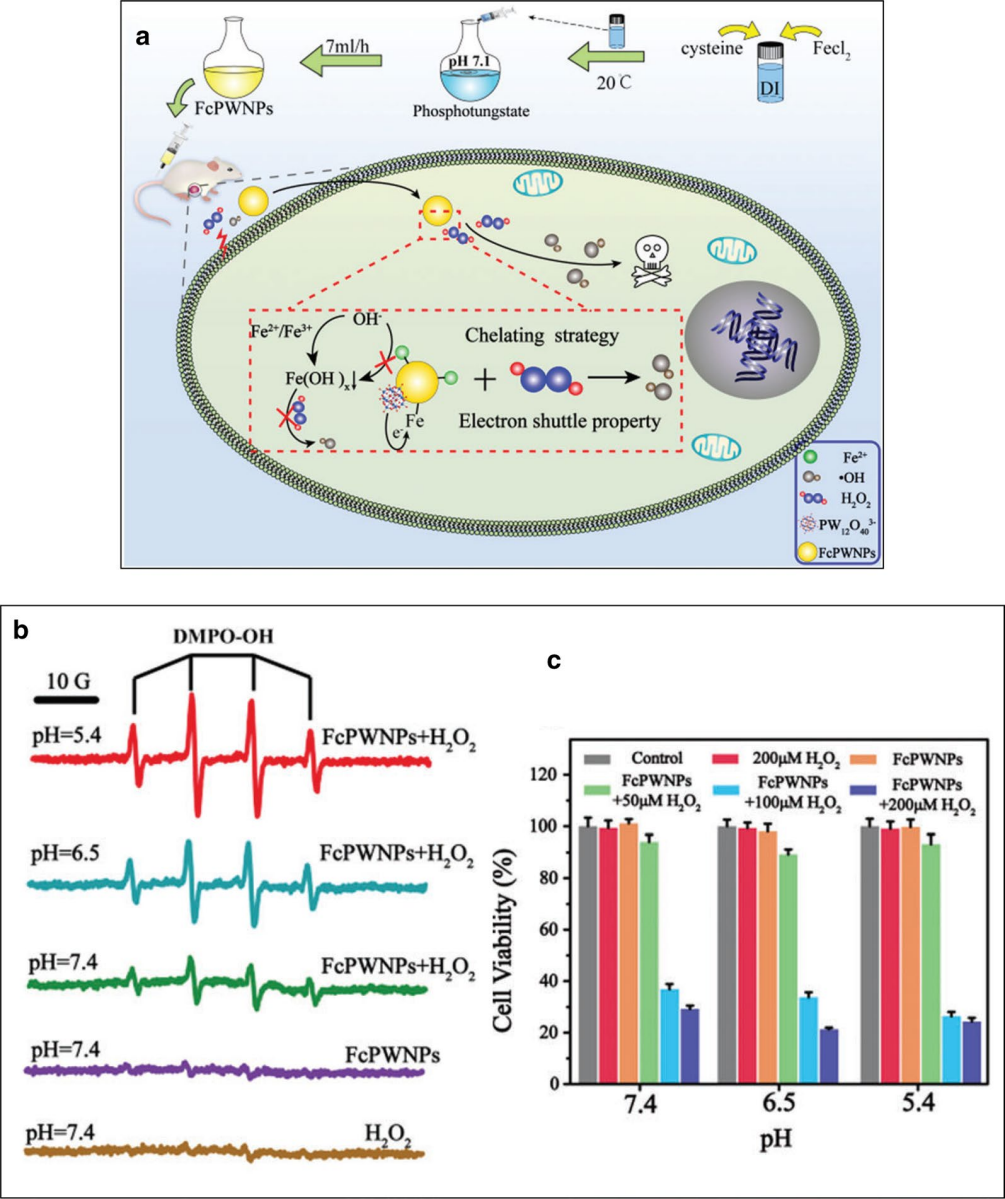
**a** Schematic illustrating the synthesis process and mechanism for FcPWNP mediated pH independent high efficiency CDT, **b** ESR spectra of different groups treated with DMPO; FcPWNPs: Fe concentration of 80 ppm, and H_2_O_2_: 1 mM. **c** The growth inhibition effect on 4T1 cells of different groups after 24 h of incubation. (Fe element: 40 ppm, H_2_O_2_: 200, 100 and 50 mM, pH 7.4, 6.5 and 5.4). (n = 6, mean ± SD)

Until now, almost all developed CDT agents are acidity dependent (optimum Fenton reaction pH: 2–4) and a few studies have been carried out to develop wide pH range-responsive CDT agents [111]. However, the neutral-pH conditions at the solid tumor surface resulting from abundant vessel distribution and sufficient oxygen supply undo the effect of acidity-activated nanoagents and even induce tumor recurrence and metastasis after treatment [112, 113]. Zhaoʼs group synthesized Ferrous-cysteine–phosphotungstate nanoagent for enhanced cancer chemodynamic therapy that breaks through the limitation of a neutral pH [114]. The advantages from the addition of phosphotungstate and cysteine to formation of a Fe^3+^ chelating complex inhibited the formation of inert Fe(OH)x, and accelerate electron transfer between ferric and ferrous ions, respectively (Fig. 11a).

To investigate the OH· generation ability of the ferrouscysteine–phosphotungstate nanoparticles (FcPWNPs), electron spin resonance (ESR) spectroscopy is conducted. As can be seen in Fig. 11b, FcPWNPs show representative hydroxyl radicals with pH-dependent tendency. The strong cytotoxicity against cancer cells with an H_2_O_2_ dose-dependent tendency was achieved inspired by the high OH· production performance across a wide pH range, (Fig. 11c).

**Fig. 12:**
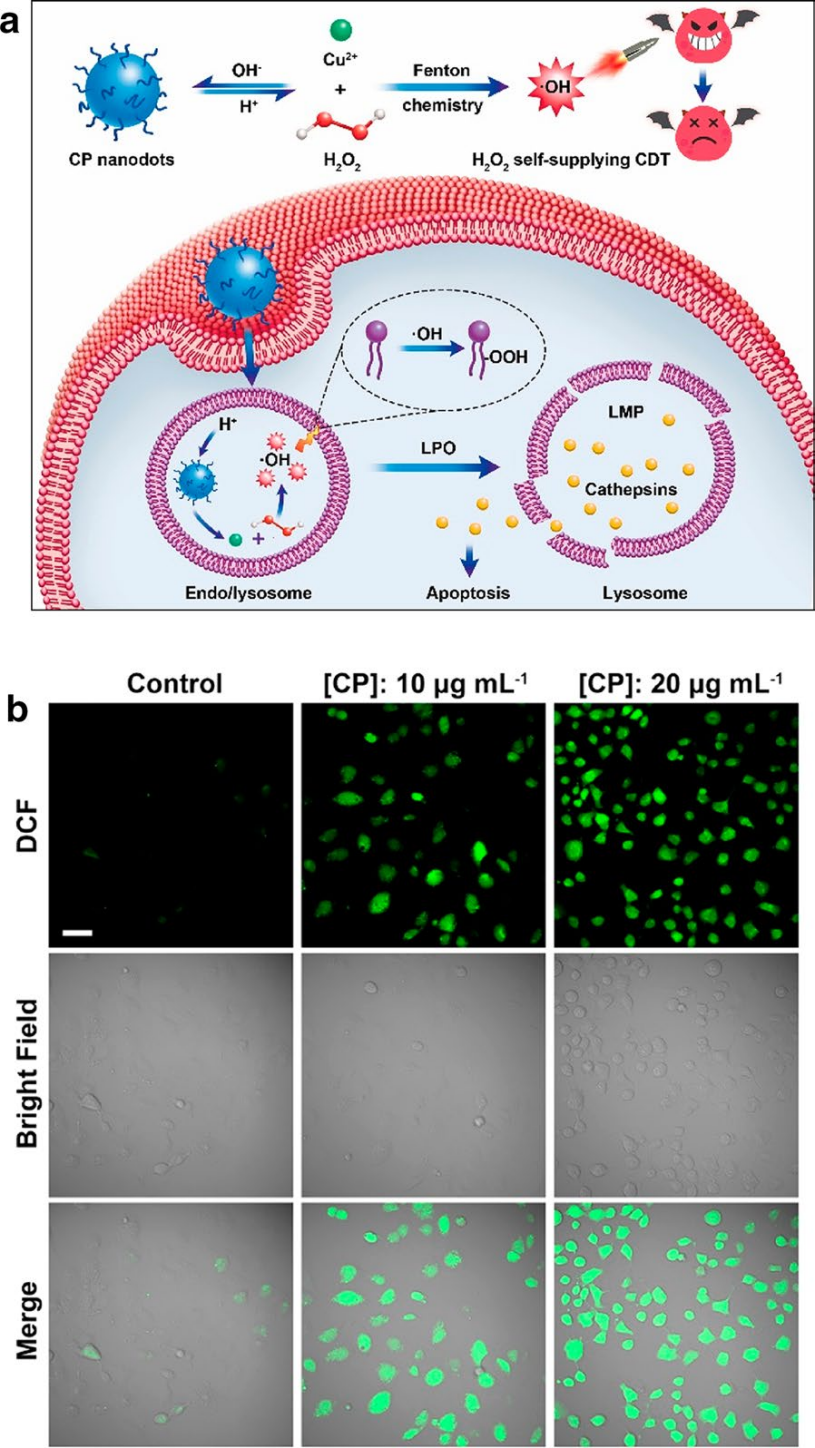
**a** Formation of CP Nanodots for H_2_O_2_ Self-Supplying CDT, **b** Fluorescence images of DCFH-DA-stained U87MG cancer cells after exposure to different concentrations of CP nanodots for 4 h. The scale bar represents 50 μm

Although, compared with normal cells, many types of tumor cells have higher intracellular H_2_O_2_ levels, the endogenously generated H_2_O_2_ is still inadequate to obtain improved CDT efficacy [115]. Thereafter, their anticancer efficiency could be enhanced by the introduction of H_2_O_2_-supplementing functionality into CDT agents. Lin et al. developed copper peroxide nanodots for H_2_O_2_ self-supplying chemodynamic therapy [116]. Copper peroxide CP nanodots were prepared through the binding of H_2_O_2_ to Cu^2+^ in the presence of poly (vinylpyrrolidone) (PVP) as stabilizer at room temperature. This Fenton-type peroxide nanomaterial utilized as an activatable agent for enhanced CDT by self-supplying H_2_O_2_ (Fig. 12a). Upon acid treatment, the reversible decomposition of CP nanodots into Fenton catalytic Cu^2+^ and H_2_O_2_ occurred.

2′,7′-dichlorofluorescin diacetate (DCFHDA), the fluorescent ROS indicator, was applied to evaluate the production of OH· by CP nanodots at the cellular level. Deacetylation of DCFH-DA and formation of nonfluorescent DCFH was accomplished by intracellular esterases, which can be oxidized by ROS and then emits green fluorescence [117]. It was shown in Fig. 12.B that U87MG cancer cells incubated with CP nanodots exhibited significantly higher green fluorescence than untreated control cells, indicating the ability of CP nanodots to generate OH· within tumor cells.

**Fig. 13:**
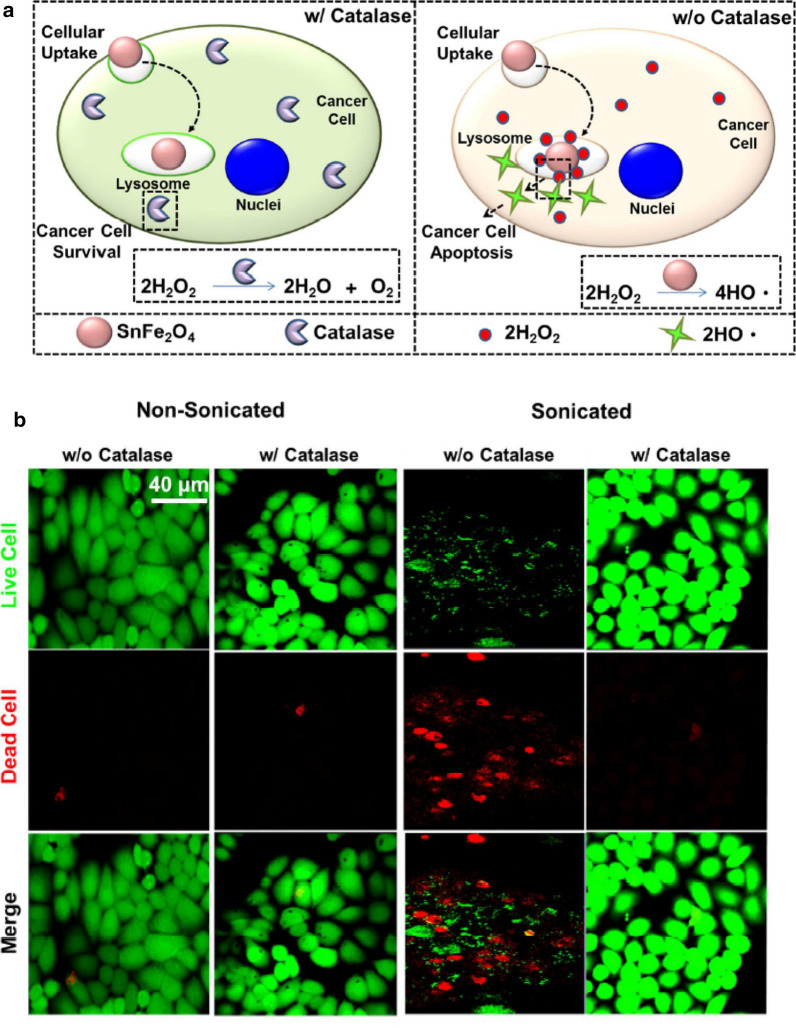
**a** Illustration showing internalized SnFe_2_O_4_ nanocrystals performing cytotoxic effect on cancer cells intracellularly and non-cytotoxic effect on cancer cells in presence of catalase. **b** Fluorescent images of slices co-stained with LIVE(green)/DEAD(red), viability/cytotoxicity assay kit for test cells

Therapeutic selectivity, characteristic differences between healthy and cancer cells, is one of the critical factors in development of cancer therapies. Due to rapidly proliferating, cancer cells have high H_2_O_2_ levels with a low catalase level in comparison with normal cells. In this regard, SnFe_2_O_4_ nanocrystals were employed for selective killing of lung cancer cells by catalase-modulating heterogeneous Fenton reaction [118]. A working mechanism of this developed assay is the inhibition of the heterogeneous Fenton reaction in normal cells with catalase through decomposition of H_2_O_2_ (Fig. 13a). Furthermore, the sonicated SnFe_2_O_4_ nanocrystals demonstrated much higher efficiency than the non-sonicated nanocrystals to produce the hydroxyl radicals. Therefore, the sonicated SnFe_2_O_4_ nanocrystals in presence of catalase exhibited low cell viability compared with test cells treated with non-sonicated nanocrystals without catalase (Fig. 13b).

**Fig. 14:**
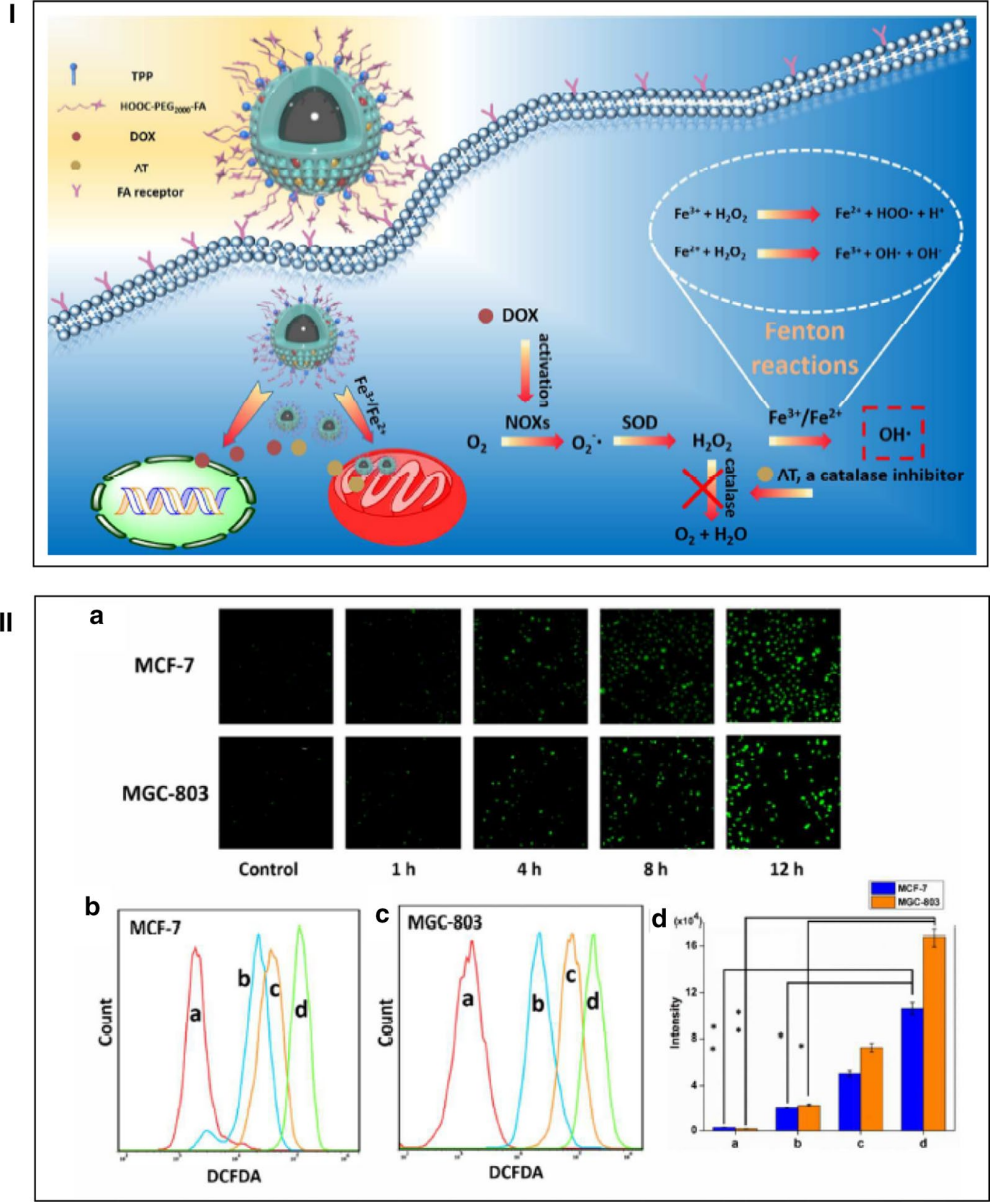
**I** The DOX/AT loaded Fe3O4@MSN-TPP/PEG-FA accumulated in the tumor cells by recognition of folate receptors and further targeted to mitochondria by TPP-mediated effect. In this study, excessively toxic OH· which could induce cell death were generated by a series of biochemical reactions. **II** Evaluation of ROS generating capability of DOX/AT-loaded Fe_3_O_4_@MSN-TPP/PEG-FA in vitro. (A) CLSM images of MCF-7 and MGC-803 cells incubated with DOX/AT-loaded Fe_3_O_4_@MSN-TPP/PEG-FA for different incubation time. Flow cytometry assay ROS level of MCF-7 cells (B) and MGC-803 cells (C). a, control group; b, DOX/AT-loaded MSN-TPP/PEG-FA; c, DOX/AT-loaded Fe_3_O_4_@MSN; d, DOX/AT-loaded Fe_3_O_4_@MSN-TPP/PEG-FA. (D) The analysis of flow cytometry. (n = 3). *P < 0.05 and **P < 0.01

In cancer therapy, DOX can activate nicotinamide adenine dinucleotide phosphate (NADPH) oxidases (NOXs) for transportation of electrons across the membrane. The activated NOXs can catalyze NADPH into NADP^+^ along with the release of electrons. Thus, the oxygen captures the electrons to produce the superoxide anion radical (O_2_^−^·) and afterward product the H_2_O_2_ by disproportionation reaction with superoxide dismutase (SOD) enzyme [119–121]. In Fenton reactions, iron oxide core–shell mesoporous silica (Fe_3_O_4_@MSN) nanocarrier promoted oxygen species levels reactive for cancer therapy [122]. Fe_3_O_4_@MSN-TPP/PEG-FA was formed by conjugation of Fe_3_O_4_@MSN with folate (PEG-FA) and mitochondrial targeting triphenylphosphonium (TPP). Then, Fe_3_O_4_@MSN-TPP/PEG-FA encapsulated doxorubicin (DOX) and 3-amino-1,2,4-triazole (AT) for cancer therapy (Fig. 14I). AT, as a catalase inhibitor inhibits the catalase activity to save the production of H_2_O_2_. The assessment of ROS level induced by DOX/AT-loaded Fe_3_O_4_@MSN-TPP/PEG-FA in MCF-7 and MGC-803 cells showed that green fluorescence was gradually improved after different incubation times (Fig. 14II). The results proved the significant elevation of the intracellular ROS level stimulated by DOX/AT-loaded Fe_3_O_4_@MSN-TPP/PEG-FA.

## Photodynamic therapy (PDT)

**Fig. 15:**
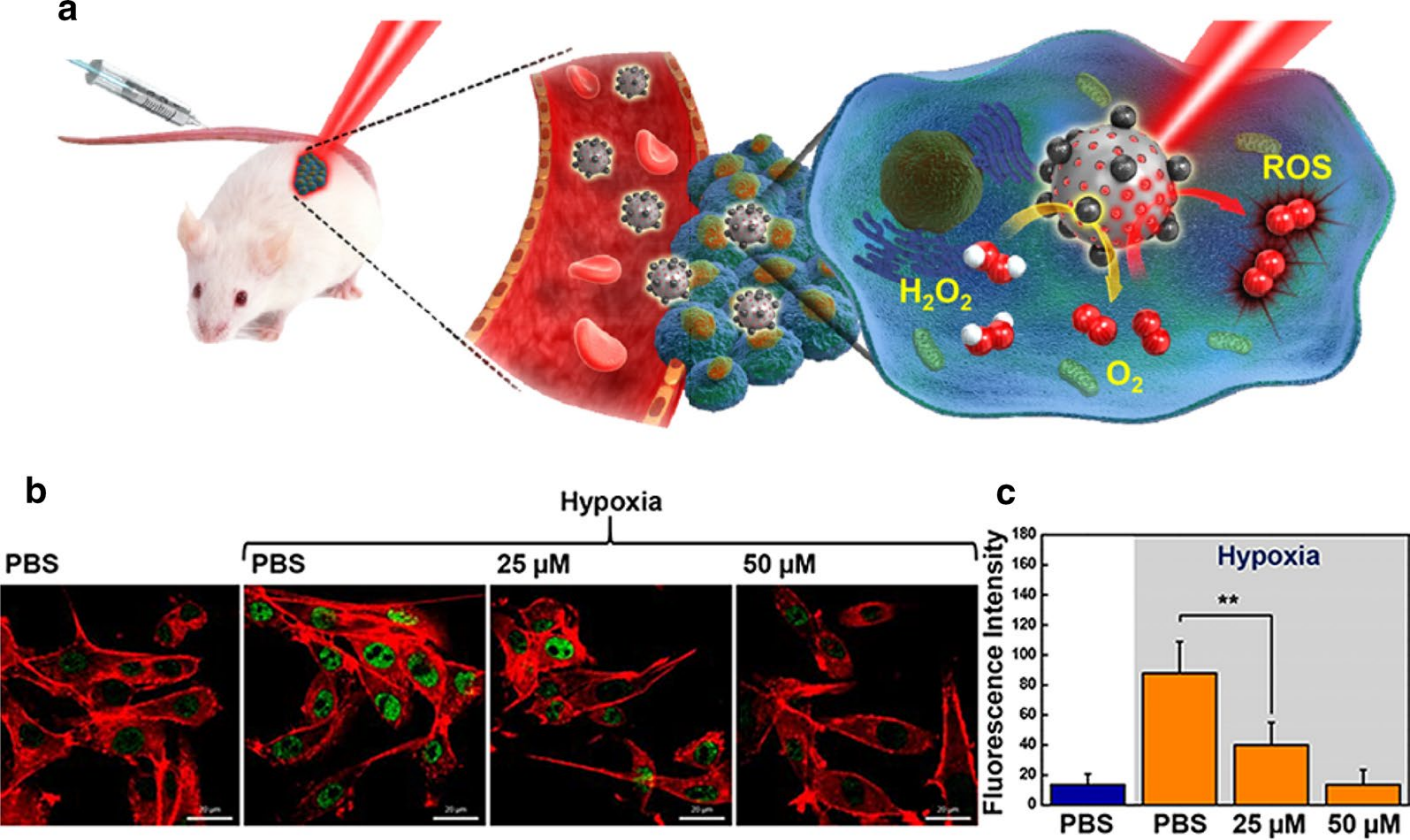
**a** Schematic illustration of MFMSNs. **b** CLSM images of HIF-1α (green) and F-actin (red) in cells incubated with MFMSN under normoxic or hypoxic condition, and **c** corresponding fluorescence intensity (n = 3). Scale bar, 20 μm. **P < 0.01

Photodynamic therapy (PDT) is the most versatile minimal invasive manner of cancer therapy, which involves the production of cytotoxic reactive oxygen species (ROS) by light activation of photosensitizers [123, 124]. The produced ROS induce cell apoptosis or necrosis, microvascular damage and immune responses. Aggressive proliferation of cancer cells and an insufficient blood supply in tumors decrease O_2_ concentration. The O_2_ deficiency in tumors leads to a significantly reduced antitumor efficacy of PDT [125–127]. In PDT, O_2_ employs to produce ROS, so, hypoxia obviously arises during PDT. Hypoxia, yielded from the imbalance between oxygen supply and consumption, is a great indicator of cancer progression. Since, O_2_ is a necessary component in PDT and hypoxia prevents effective cancer treatments [128, 129]. In view of this, tremendous attention has been attracted to overcome tumor hypoxia. Recently, various nanomaterials with catalase like activity, have been employed to catalytically generate O_2_ to mitigate cancer hypoxia. For example, manganese ferrite nanoparticle-anchored mesoporous silica nanoparticles to eliminate hypoxia and efficient photodynamic therapy [130]. Manganese ferrite nanoparticles (MFNs) act as a Fenton catalyst for decomposition of H_2_O_2_ and continuous O_2_ generation. The level of hypoxia can be examined based on HIF-1α amounts, because hypoxia-inducible factor (HIF-1α) protein is adjusted under hypoxic condition [131]. As can be seen in Fig. 15, when the cancer cells were treated with manganese ferrite nanoparticle-anchored mesoporous silica nanoparticles (MFMSNs), the fluorescence intensity of HIF-1α has decreased in concentration dependent manner, suggesting the capability of MFMSNs to reducing hypoxia via O_2_ production after cellular uptake.

**Fig. 16:**
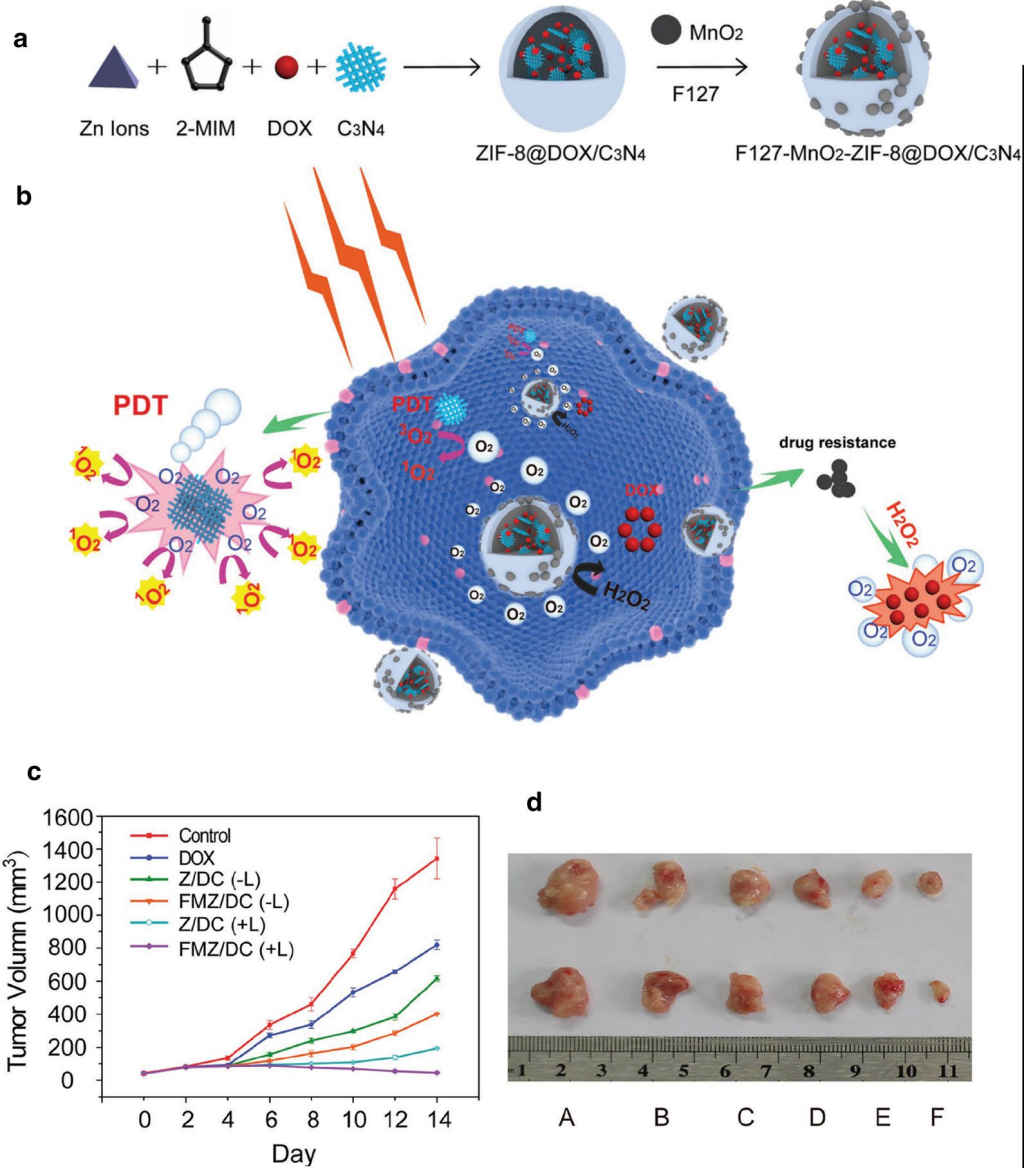
**a** Schematic illustration of the fabrication of FMZ/DC nanocomposites. The diagram is not drawn to scale. **b** Schematic illustration of FMZ/DC with oxygen generation enhancing the chemo-photodynamic therapy under 660 nm light irradiation. In vivo combination therapy of FMZ/DC by intravenous administration into a 4T1 tumor mouse model. **c** Tumor growth curves of different groups of 4T1 tumor-bearing mice. The laser irradiation (+ L) was carried out under 660 nm light at the power density of 5 mW cm − 2 for 30 min. Error bars were based on five mice in each group. **d** Images of tumors collected from different groups of mice 14 d after different treatment: **a** control; **B** DOX; C, Z/DC (− L); **d** FMZ/DC (− L); **e** Z/DC (+ L); **f** FMZ/DC (+ L)

In another study, Zhang et al. used MnO_2_ nanodots to promote dissolved oxygen concentration and overcome hypoxia limitations [132]. The PDT nanoplatform is fabricated by one-pot encapsulating g-C_3_N_4_ and DOX in ZIF-8, then loading MnO_2_ nanodots and surface-modifying F127 (F127-MnO2-ZIF@DOX/C3N4, donated as FMZ/DC). F127 with excellent biocompatibility and amphiphilic nature is chosen as a stabilizing agent. Encapsulation into pH-dependent ZIF-8 carrier reduces the side effects of DOX induced by nonspecific drug release. In addition, g-C3N4, a prominent visible-light photocatalyst, could efficiently generate ROS and kill cancer cells (Fig. 16a, b). The efficacy of the tested materials to the living body was confirmed by measuring the tumor sizes of mice during the healing process (Fig. 16c, d).

**Fig. 17:**
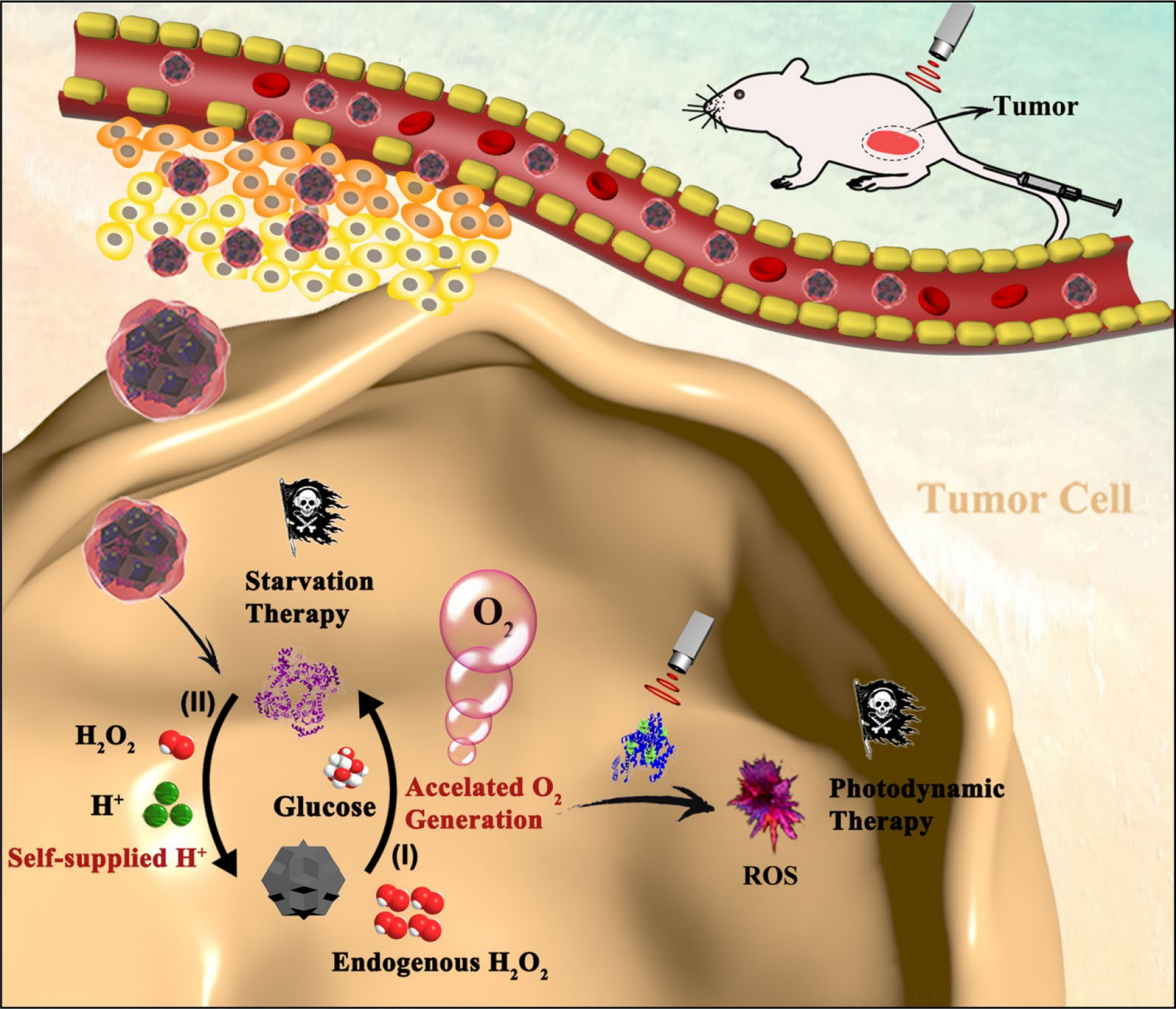
The scheme of MnO_2_-GOx hybrid achieving self-supplied H^+^ and accelerating O_2_ generation for alleviating tumor hypoxia and enhancing PDT and starvation therapy against hypoxic tumors

MnO_2_ biomimetic nanozyme could be integrated glucose oxidase (GOx) enzyme for improved starvation and photodynamic therapy [133]. GOx can oxidize glucose to gluconic acid and H_2_O_2_, which is capable of tumor starvation therapy. Meanwhile, MnO_2_ accelerated O_2_ production with the aid of a large amount of H^+^ from oxidation product gluconic acid. This O_2_ supply decreases tumor hypoxia and promotes PDT effectiveness (Fig. 17).

**Fig. 18:**
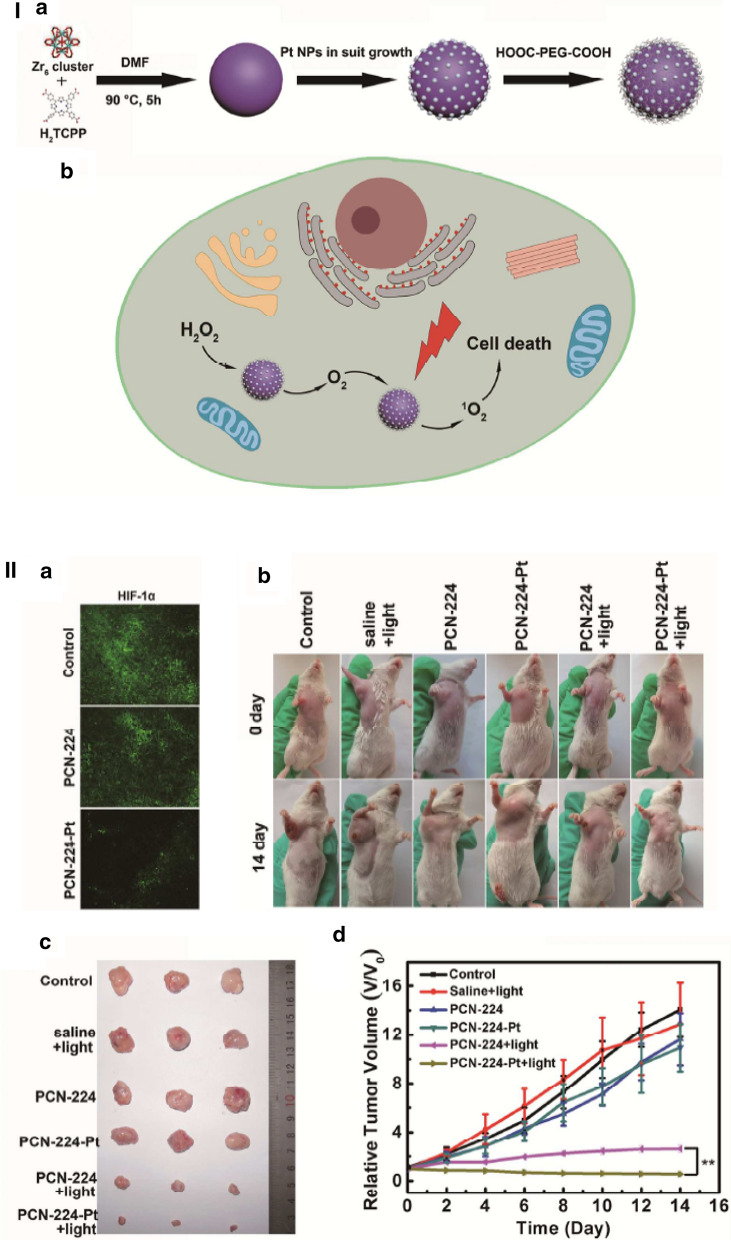
**I** Schematic illustration of (A) the preparation process of PCN-224-Pt and (B) the use of PCN-224-Pt for enhanced photodynamic therapy, **II** Photodynamic therapy of PCN-224-Pt by intratumoral injection in a subcutaneous tumor model. A) HIF-1α staining of tumor tissues collected from mice in different groups. B) Photographs of the H22 tumor-bearing mice before treatment and on day 14 after the various treatments. C) Representative photographs of the tumor dissection. D) Relative tumor volume after various treatments indicated. Asterisks indicate significant differences (*P < 0.05, **P < 0.01, ***P < 0.001)

To achieve more stability and hinder the aggregation of small size mimicking enzyme nanoparticles, Zhangʼs group decorated Pt nanozymes on photosensitizers integrated MOFs for enhanced photodynamic therapy [134]. Pt nanoparticles with catalase like activity were decorated on porous coordination network-224 (PCN-224). The PCN-224-Pt could assist the formation of ^1^O_2_ in hypoxic tumor site via decomposition of H_2_O_2_ for producing O_2_, which could be employed for enhanced photodynamic therapy (Fig. 18I). Decreased in immonufluorescence intensity of HIF-1α for treated tumor slices with PCN-224-Pt indicated remove the hypoxia limitation by Pt NPs on PCN-224-Pt (Fig. 1). In the case of tumors of mice injected with PCN-224, tumor growth was completely inhibited after irradiation treatment (Fig. 18II).

**Fig. 19:**
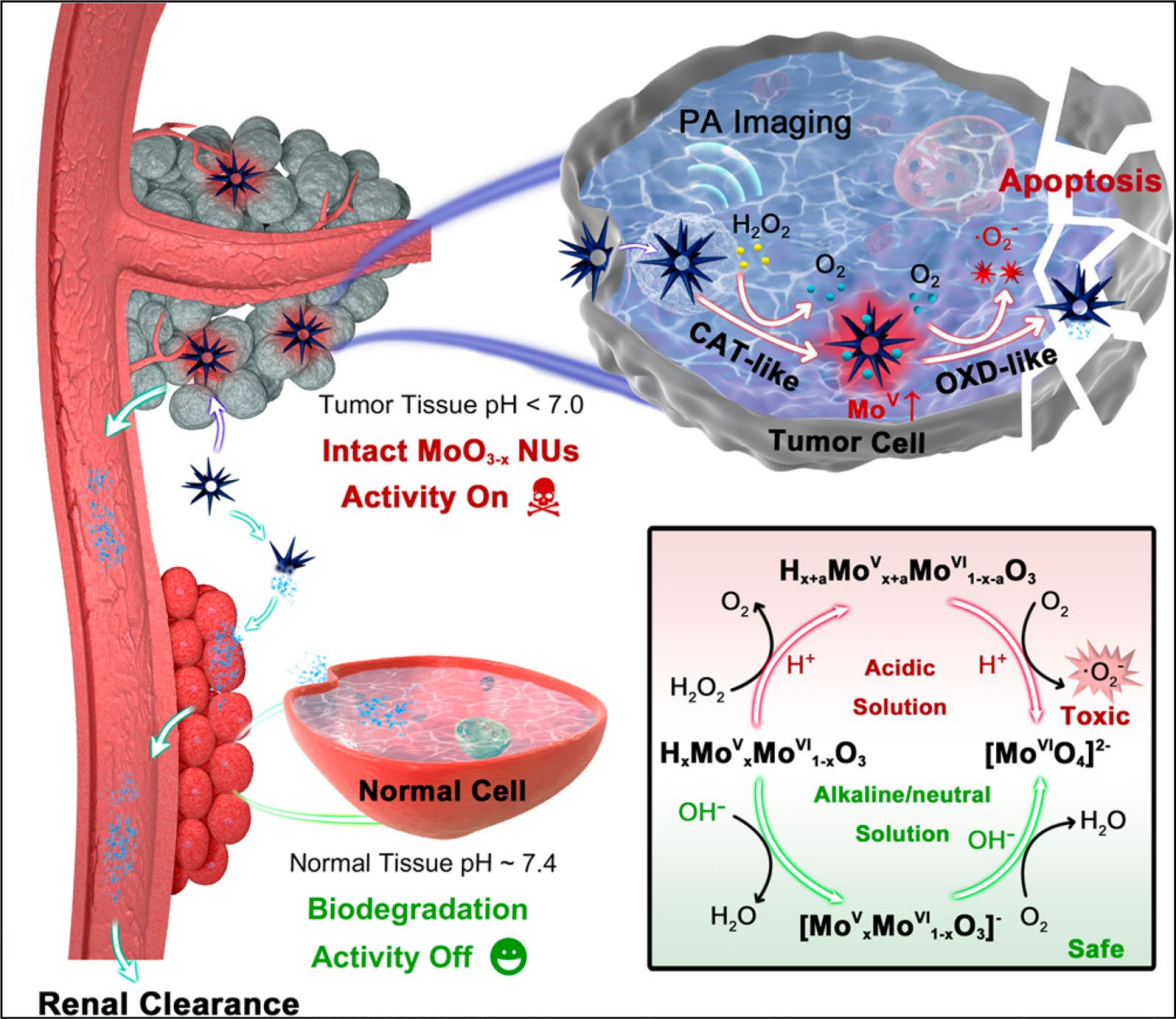
Schematic Illustration of Biodegradation-Medicated Enzymatic Activity-Tunable Molybdenum Oxide Nanourchins (MoO3 − x NUs) with the Highly Specific Toxicity to Tumor Tissues via a Multienzyme Stepwise Cascade Catalysis in Acidic Tumor Microenvironment

It is reported that the activities of enzyme mimics are correlated with their nanostructures [135]. For instance, MoO_3_ − x nanourchins (NUs) exhibited a structure-dependent enzymatic activity with therapeutic effect in tumor microenvironment via cascade catalytic reactions [136]. In this design, MoO_3_ − x NUs possess high proportion of active Mo^V^ atoms and large active surface area, induce catalase (CAT)-like activity to produce a large amount of O_2_ for subsequent oxidase (OXD)-like reactivity (Fig. 19). The reactivity of MoO_3_ − x NUs in acidic PBS is much higher than that in neutral or alkaline; thus, MoO_3_ − x NUs would rapidly lose the enzymatic activity and leave normal tissues unharmed in a physiological environment (pH ∼ 7.4).

## Photothermal therapy (PTT)

Photothermal therapy (PTT) is a treatment modality with minimum side effects, which involves the artificial elevation of the tissue temperature. PTT agents capture near-infrared (NIR) light and convert it into heat, causing tumor cells apoptosis [137–139]. Without any laser exposure,

PTT agents are nontoxic and relatively safe to cells. In compare with traditional tumor treatment models including radiotherapy, surgery, and chemotherapy, PTT is attractive because of certain advantages, such as reduced invasiveness and high specificity [140, 141]. Nevertheless, tumor cells usually cannot be completely killed by photothermal treatment alone, finally resulting in tumor recurrence [142]. Thus, to further enhance the therapeutic performance of PTT, the enzyme-mimicking performance of nanozymes can be employed.

**Fig. 20:**
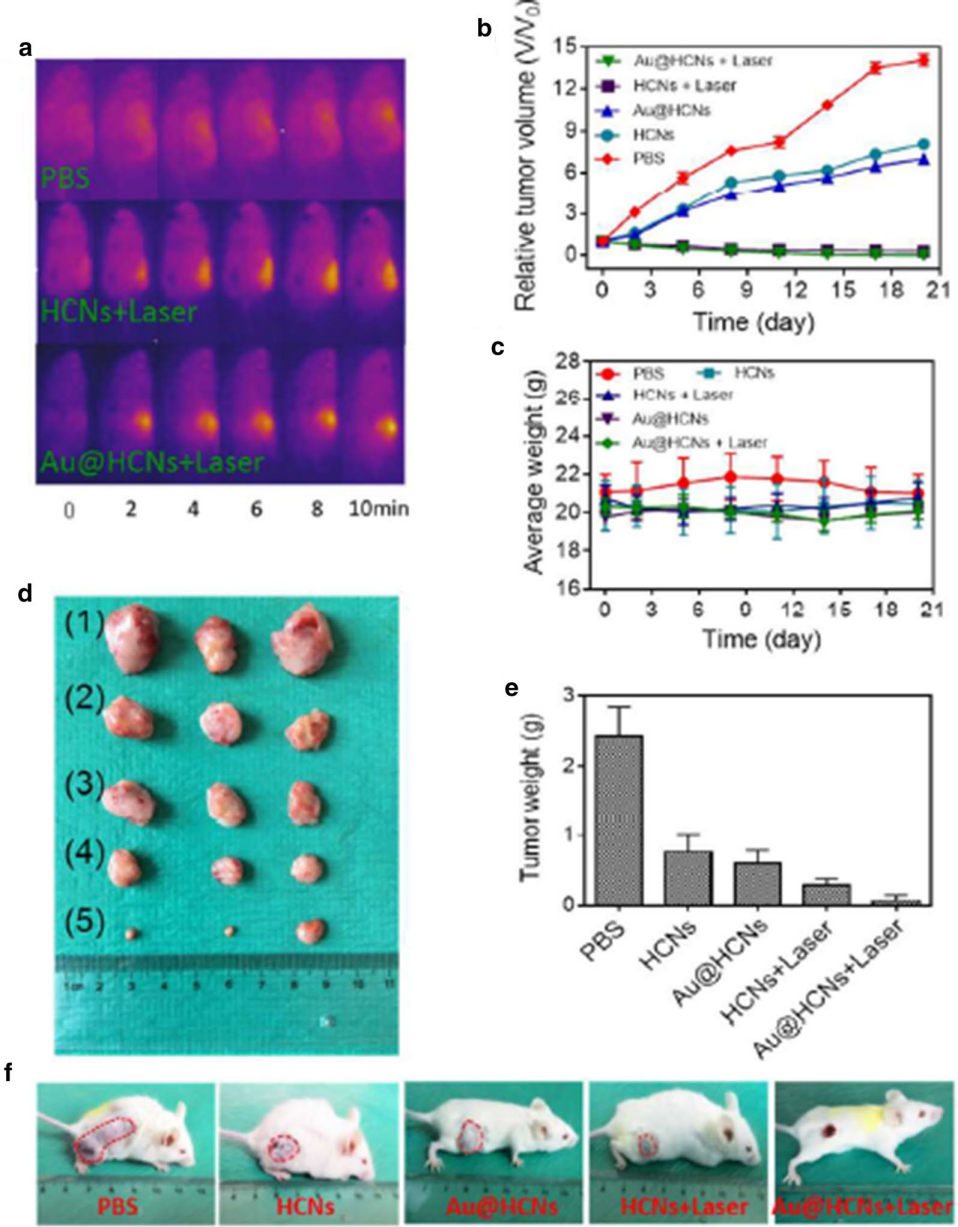
In vivo catalytic-photothermal therapy of CT26 tumor bearing mice. **a** IR thermal images of CT26 tumor-bear mice with the NIR laser irradiation (808 nm, 2.0 W/cm^2^, 10 min) after intravenous injection with PBS, HCNs and Au@HCNs. **b** Tumor growth curves of different groups after treatment. **c** The body weight after various treatments during 21 days. **d** Photos of tumors from (1) control, (2) HCNs, (3) Au@HCNs, (4) HCNs + Laser, (5) Au@HCNs + Laser. **e** The Tumor weight after 21 days of treatment. **f** Representative photos of tumors on mice after various treatments after 21 day

Au nanoparticles (Au NPs) are one of the most widely studied photothermal agents owing to their effective local heating upon excitation of surface plasmon oscillations. Besides, many studies displayed that Au NPs possess enzyme-like activities [143]. In this regard, Fanʼs group utilized yolk-shell gold@carbon nanozymes Tumor catalytic-photothermal therapy [144]. A hollow carbon nanospheres with porous shell (Au@HCNs) exhibited high oxidase-like and peroxidase-like activity enzyme activities. Meanwhile, Au@HCNs outstanding near-infrared light (NIR) absorbing agents for convert light into heat for tumor photothermal therapy (PTT). The enzyme-mimicking functions significantly improved by the photothermal effect, leading to large amounts production of ROS to destruct cancer cells. Tumor mice have been exposed with different groups of agents with or without NIR irradiation. The results showed that the tumors treated with Au@HCNs under 808-nm laser irradiation were completely destroyed without recurrence during the treatment (Fig. 20).

Nanoceria (NCeO_2_) decorated flower-like MoS_2_ nanoflakes reported as a nanozyme for cancer photothermal therapy (PTT) [145]. Polyethylenimine (PEI) coated flower-like MoS_2_ nanoflakes surface decorated with cerium oxide NPs to formation of NCeO_2_-PEI-MoS_2_. The NCeO_2_ decoration considerably enhanced the photoconversion effectiveness (PCEs) of MoS_2_ nanoflakes. The different effects of NCeO_2_-PEI-MoS_2_ nanoflakes on cancer and normal cells were due to multi-enzyme mimics of NCeO_2_. Normal cells protect against oxidative damage via neutralization of free superoxide radicals and hydrogen peroxide by superoxide dismutase (SOD) and catalase enzyme mimic to decompose H_2_O_2_ into water and O_2_. Although, in acidic cancer cell NCeO_2_ work as a Fenton-like catalyst that dismutates H_2_O_2_ to OH^·^. Next, O_2_^−·^ and H_2_O_2_ are formed by dismutating CO_2_ or ordinary molecular oxygen (O_2_). The formed.

**Fig. 21:**
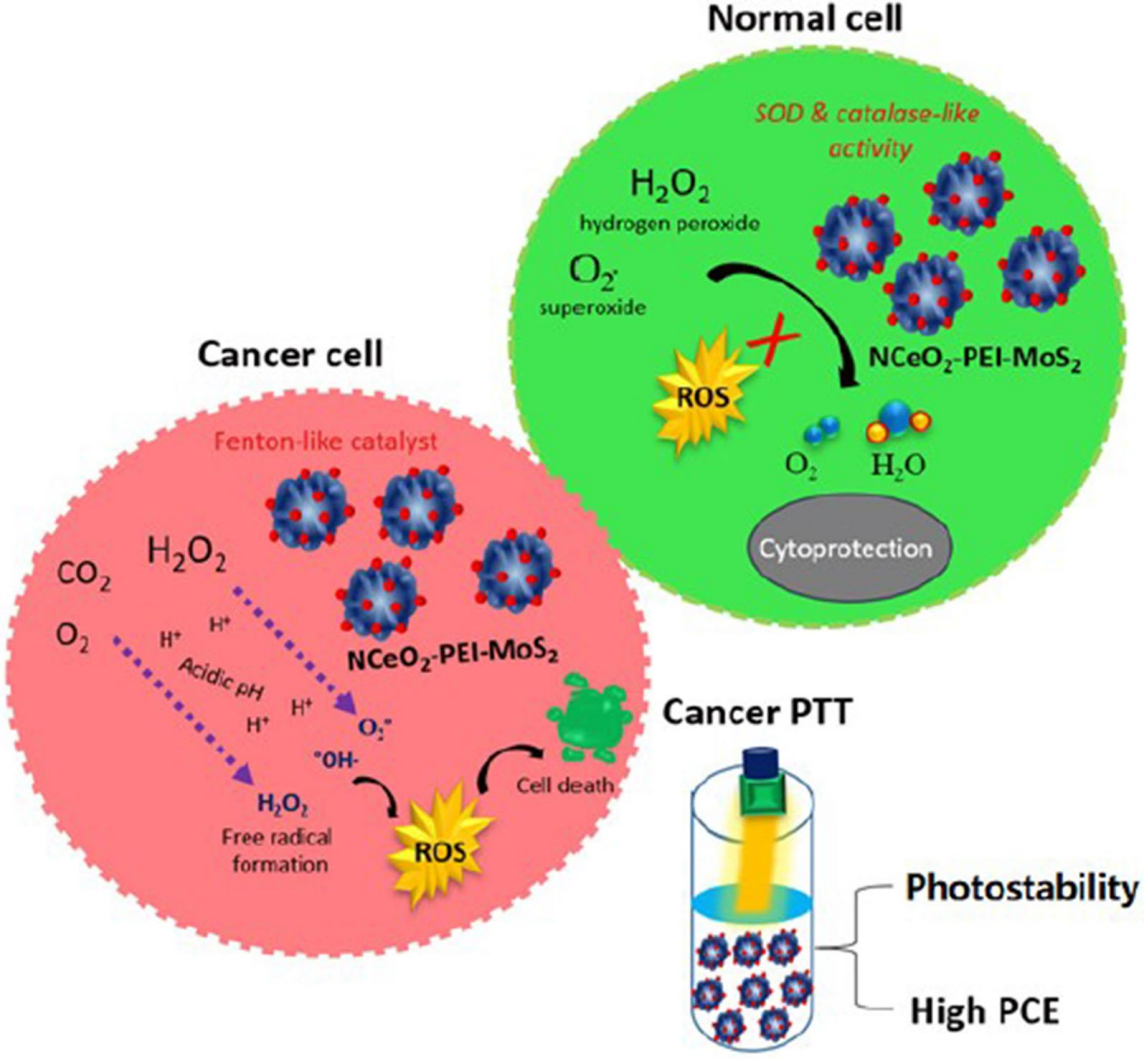
Schematic illustration of the use of NCeO_2_-PEI-MoS_2_ for selective enhanced photodynamic therapy

ROS species (OH·, O_2−_· and H_2_O_2_) induce oxidative stress leading to cell death or apoptosis in cancer cells (Fig. 21).

## Sonodynamic therapy (SDT)

**Fig. 22:**
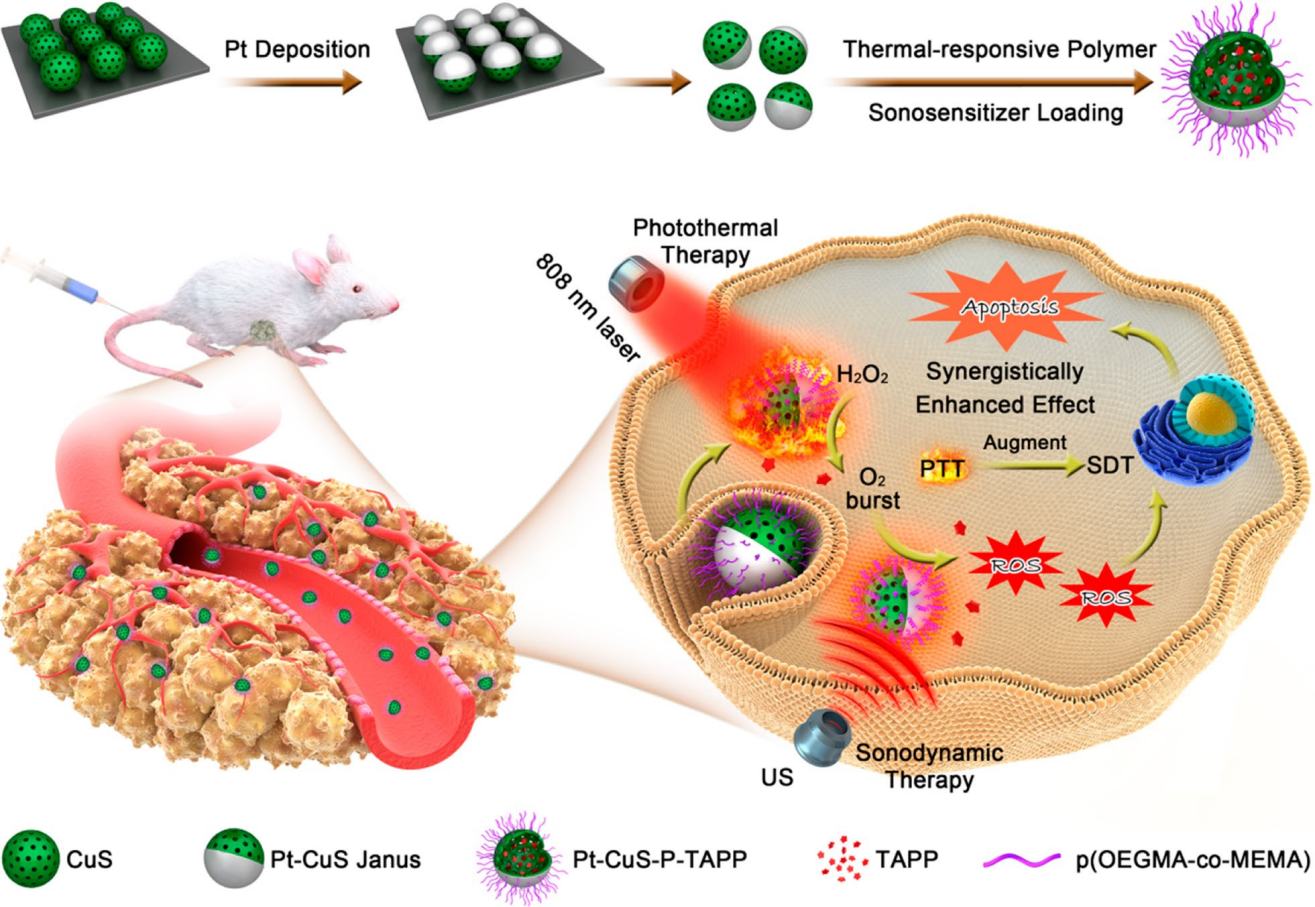
Schematic illustration of the main synthesis procedures and antitumor mechanism of Pt-CuS NPs

Ultrasound (US) can penetrate biological tissues, capable of activating sonosensitizers to generate toxic ROS molecules for cancer therapy modality named sonodynamic therapy (SDT). SDT can obviate the severe issue of low tissue-penetrating depth of traditional phototriggered therapies, but the SDT efficiency is still not satisfactory in battling cancer [146–148]. In SDT, ultrasound (US) can trigger sonosensitizers to produce ROS, bubbles, cavitation and hyperthermia. it has a good therapeutic effect on the treatment of deep malignant tumors. SDT is a selective method for treatment of deep malignant tumors, because US can precisely focus on the tumor region, target to activate sonosensitizers, and minimize the damage to the adjacent normal organs and tissues [146, 149]. The solid tumor microenvironment (TME) appears when there is critical hypoxia because of O_2_ consumption during SDT [150]. Thereby, enzyme mimic nanomaterials can also work as the synergistic agents to boost the therapy efficiency by alleviating tumor hypoxia. Nanoenzymes can convert the tumor-overexpressed hydrogen peroxide (H_2_O_2_) molecules into oxygen and enhancing the tumor oxygen level to boost SDT-induced ROS production. Liang et al. employed hollow Pt-CuS janus architecture for synergistic catalysis-enhanced sonodynamic and photothermal cancer therapy [151]. Sonosensitizer molecules (tetra-(4-aminophenyl) porphyrin, TAPP) loaded on inner cavities of hollow CuS to fulfillment SDT. Metallic Pt with enzyme like-activity catalyzed decomposition of endogenous overexpressed H_2_O_2_ to produce O_2_ and facilitates SDT efficacy (Fig. 22). Nanozymes can act as a carrier effectively deliver a sonosensitizer to the lesions and also provide a sonosensitizer with rich oxygen by their enzyme activities. It was found that the modification of a sonosensitizer onto Pd@Pt could significantly block the catalase-like activity of Pd@Pt, whereas upon US irradiation, the nanozyme activity was effectively recovered to catalyze oxygen generation [152]. Such “blocking and activating” enzyme activity decreases the potential toxicity and side effects of nanozymes on normal tissues and helps realize controllable, active, and disease-loci-specific nanozyme activity behavior.

## Antibacterial applications

**Fig. 23:**
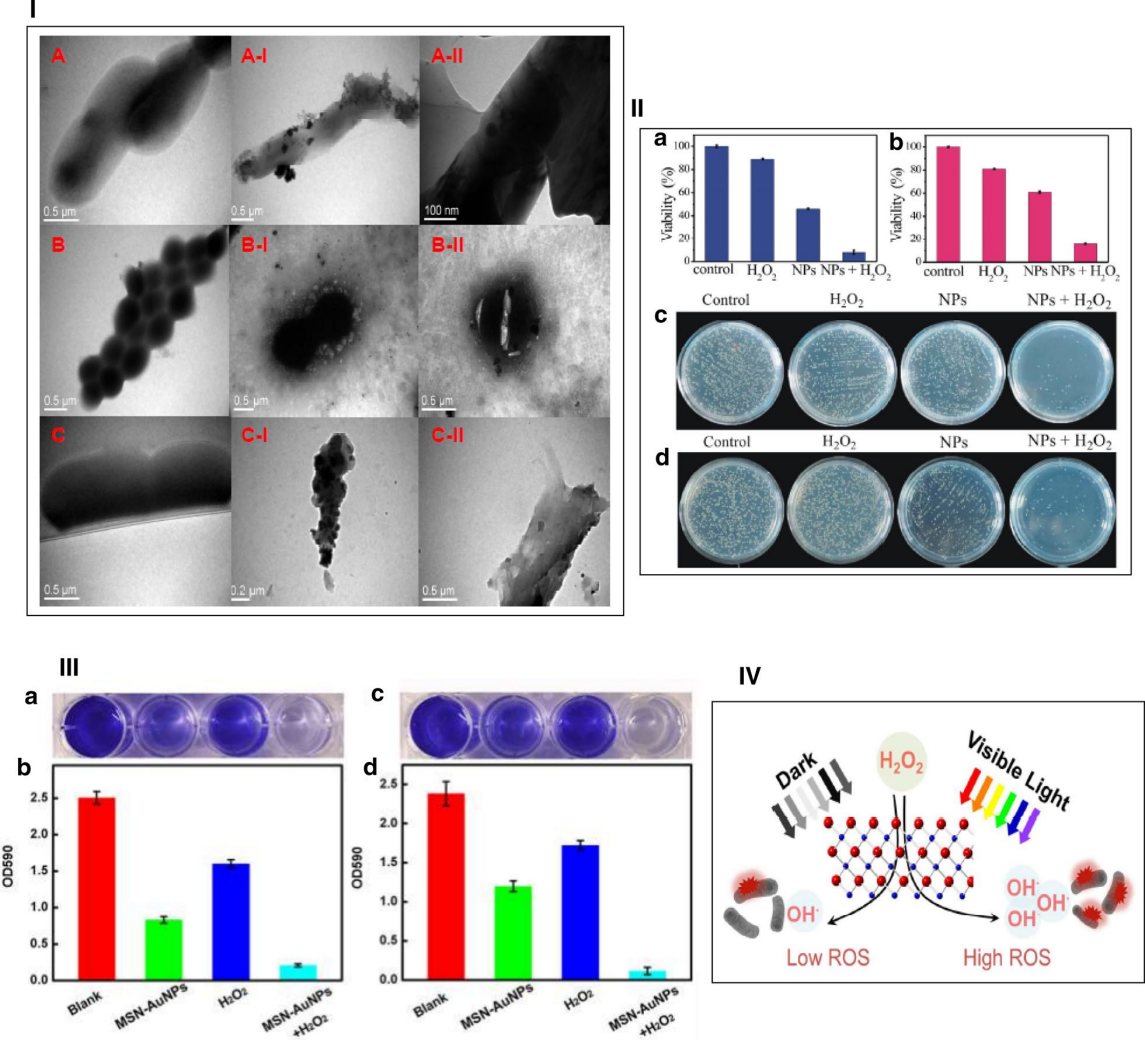
**I** TEM images of *E. coli* cells (A), *S. aureus* cells (B), and drug resistant *E.coli* (C), before (A, B and C) and after (A-I, A-II, B-I, B-II, C-I and C-II) treatment with 20 μg/mL GQD-AgNP hybrids, **II** Cell viability of (a) *E. coli* and (b) *S. aureus*; the plate samples showing colonies of (c) *E. coli* and (d) *S. aureus*, **III** (a,b) The effect of the MSN-AuNPs based antibacterial system on the biofilm destruction of B. subtilis. (a) Pictures of crystal-violet-stained the remaining biofilms. (b) The remaining biofilms were quantified by crystal violet staining. (c,d) The effect of the MSN-AuNPs based antibacterial system on the biofilm formation of B. subtilis. (c) Pictures of crystal-violet-stained the generated biofilms. (d) Quantification of the generated biofilms by crystal violet staining, **IV** Schematic illustration of nanozyme-catalysed antibacterial performance of CuO NRs

Infectious diseases caused by bacteria are the most growing global health problem, infecting millions of people every year [153]. Until now, a number of antibacterial materials, such as antibiotics, quaternary ammonium ion, metal ions, and biocides have been developed to counter the growth of dangerous bacteria [154, 155]. However, owing to high cost of the above materials, antibiotic resistance, and complex chemical processing, the provision of alternative antimicrobials is of particular importance [156]. It was found that artificial enzyme mimics are able to function as an antimicrobial against both Gram-positive and Gram-negative bacteria via increasing the transformation of H_2_O_2_ into ROS [157, 158]. Chen and coworkers have found that graphene quantum dot/silver nanoparticle (GQD/AgNP) hybrids with peroxidase and oxidase like functions demonstrate high antibacterial properties against both Gram-negative and Gram-positive bacteria via using oxygen instead of H_2_O_2_ [159]. The GQD/AgNP hybrids could induce release of ROS to oxidize the lipids in the cell membrane. After distribution of GQD/AgNP hybrids around the bacteria the surface morphology of the bacteria exhibited an evident conversion from a smooth cell membrane to a roughened and wrinkled appearance, denoting that the cell membrane had been destroyed and lost its original barrier action (Fig. 23I). The oxidization of the lipids in the cell membrane and disruption the cell metabolism result in bacteria death.

Cai et al. also fabricated porous Pt/Ag nanoparticles with excellent multifunctional enzyme mimic activities to exert excellent antibacterial effects [160]. The multi-catalytic capability of Pt/Ag NPs as oxidase peroxidase and catalase, results in suppression of the bacteria growth (Fig. 23II).

Bacterial biofilms are determined as groups or clusters of bacteria, surrounded by a self-produced matrix of extracellular polymeric substances forming the three-dimensional structure. Bacterial biofilms are associated with health problems and diseases including chronic infections, biofouling of implants, as well as tooth decay and biomedical devices. Biofilms are difficult to destroy, because the biofilm mode of growth exerts the protection effect [161–163]. Tao et al. have also demonstrated intrinsic oxidase and peroxidase catalytic functions of bifunctionalized mesoporous silica-supported gold nanoparticles for biofilm destruction [164]. Treatment with both MSN-AuNPs and H_2_O_2_ led to breaking down the existing biofilm (Fig. 23III).

Light-absorbing characteristics of nanomaterials have created salient opportunities in applying light to activate nanomaterials for control of biological processes. In particular, combining the use of light as a trigger with the nanozyme activity of nanomaterials offers remarkable potential to control the antibacterial property [165, 166]. Karim’s group showed the ability of visible light to work as an external trigger for controlling the antibacterial property of semiconducting CuO nanorods (NRs) [167]. In visible light illumination, the apparent binding affinity of CuO NRs to H_2_O_2_ increased by over four times in comparation with non-illuminated conditions, (Fig. 23IV). The outcome of this distinct feature is progression in the rate of ROS production, so that, antibacterial efficiency of photoilluminated CuO NRs improved by elevating the OH· radical formation even at low H_2_O_2_ concentrations.

The surface morphology of nanocomposites has a key role in adjusting their catalytic functions. Thus the enzyme-like activity of metal-based nanomaterials could be controlled by modulating their exposed facets [168, 169]. Along these lines facet-dependent of palladium (Pd) nanocrystals have been demonstrated against Gram-positive and Gram-negative bacteria [170]. The antibacterial performance of Pd nanocrystals against Gram-positive bacteria is the consequence of the extent of their enzyme-like activity, that is {100}-faceted Pd cubes with higher activities destroy bacteria more effectively than {111}-faceted Pd octahedrons. This outcome has been corroborated with the dissociative energy profiles for the O_2_ molecule on the Pd {111} and {100} surfaces. The O_2_ binding on the Pd {111} facet has adsorption energy of − 0.85 eV, whereas for the O_2_ on the Pd {100} facet, the value is − 1.40 eV, indicating that the Pd {100} facet, present in the Pd cubes, exhibits a stronger affinity for the O_2_ molecule.

## Water purification

Organic dyes are one of the widely used industrial products, which, their inseparable disposal poses serious risks to the environment [171]. Dyes generally cause water contamination and many problems to human health and environment, because they are toxic, mutagenic, carcinogenic and non-biodegradable. Therefore, the removal of dyes from wastewater is indispensable, particularly for securing aquatic life and mitigating the problem of water pollution [172, 173]. Among different removal technologies available for the removal of dye-containing wastewater, advanced oxidation processes (AOP) has been suggested as an excellent strategy [174, 175]. Fenton process as a one of the advanced oxidation technologies is a strong catalytic reaction used for environmental restoration. In Fenton chemistry, H_2_O_2_ is decomposed by soluble Fe^2+^ ions to produce highly oxidative species, i.e., hydroxyl radicals, according to the Haber–Weiss mechanism in Eq. (1) [176, 177]:

$${\text{Fe}}^{{{2} + }} + {\text{ H}}_{{2}} {\text{O}}_{{2}} \to {\text{Fe}}^{{{3} + }} + {\text{ HO}}^{ \cdot } + {\text{ OH}}^{ - }$$

The homogeneous Fenton process based on the aqueous mixture of ionic iron (Fe^2+^/Fe^3+^) and hydrogen peroxide (H_2_O_2_) suffers from sludge formation and pH limitations. Hence, much attempt has been devoted to the development of heterogeneous Fenton catalysts to address these issues [178, 179]. In that respect, iron oxide nanocomposite has attracted much interest for their applications in catalytic degradation of organic pollutants with H_2_O_2_ [180, 181]. Various forms of iron oxides such as Fe_3_O_4_, Fe_2_O_3_ and CuFe_2_O_4_ have been used as catalysts to activate H_2_O_2_ and generate ROS to remove of organic pollutants [182–184]. However, long reaction time and high concentration of H_2_O_2_ are the limitations of the H_2_O_2_-iron oxide catalytic system [185]. Therefore, the peroxidase-like activity of catalyst should be enhanced. Transition metal substituted magnetite could be introduced to improve the degradation of organic pollutants via Fenton reaction [186]. It was found that the degradation of methylene blue could be significantly improved through incorporation of niobium with magnetite catalyst. This ascribed to the generated oxygen vacancies on the surface of catalysts. Fe^2+^ cations were regenerated by introduction of Nb cations in Fenton oxidation cycle [187]. An ionothermal synthesis approach has been reported to generate Fe_3_O_4_ MNPs with unique intrinsic catalytic activity. In this synthesis strategy a deep eutectic solvent (DES) was applied for Fe^2+^/Fe^3+^ co-precipitation. As compared to Fe_3_O_4_ particles prepared in conventional commonly used solvents (water and ethyleneglycol), Fe_3_O_4_ MNPs made in DES possessed the higher activity for catalytic degradation of Rhodamine B (RhB) [188]. Utilization of some other Fenton catalyst was investigated for degradation of the organic dyes. Application of nanoceria, with excellent structural properties and high oxygen mobility, was studied for the removal of organic dyes. The proposed mechanism of Fenton-like reaction catalyzed by nanoceria is the activation of H_2_O_2_ by Ce^4+^/Ce^3+^ sites and then decompose into highly reactive hydroxyl radicals [189].

## Summery and prospect

Since the discovery of Fe_3_O_4_ nanoparticles as peroxidase mimics in 2007, nanozymes as novel emerging and rapidly growing field have gained much attention. In this regard, metal and metal oxide nanomaterials are a good candidate to replace some complicated and expensive enzymes for using them as a novel and unique techniques in various areas such as bio nanotechnology and environmental governance. In comparison with natural enzyme, nanozymes encompasses many advantages including easy preparation, excellent stability, low cost, and good durability. In this review, we have summarized the recent achievements in application of metal and metal oxide-based nanozymes, including sensing, therapeutics, antibacterial application and water treatment. Obviously substantial progress has been achieved in research field of nanozyme; however, there are still numerous challenges remain to be addressed. First, the diversity of nanozymes is very low compared to natural enzymes; in other words, though many nanomaterials have been applied to mimic natural enzymes. Currently the redox enzyme mimics are still prevailing in the peroxidase-like nanozymes. Thus, new strategies are required to design and prepare other types of nanozymes. Second, in comparison with natural enzyme, nanozymes should provide a competitive catalytic selectivity and efficiency for practical applications. The surface modifications of nanomaterials by functional groups to make the active site for substrate recognition can boost the binding affinity and specificity of nanozymes. Furthermore, designing hybrid nanomaterials with synergetic effect can help to improve their activity. Third, in general, the developed nanozymes just have one enzymatic activity. The researchers need to pay more attention to construct nanozymes which catalyze cascade reactions to mimic the complex natural enzyme systems. Forth, although nanozymes offer cost-effective methods for application in various fields, noble metal nanomaterials (Au, Pt and Pd) don't take advantage of low cost. Therefore, the efforts should be propelled to synthesis and application of non-noble nanozymes as low cost and available materials. Fifth, potential toxicities of nanozymes should be carefully considered for biomedical applications. Sixth, the current research on applications of nanozymes are mainly limited to medicine and biotechnology. Future research should be focused on widening the practical applications of nanozymes in other fields including food, industry, agriculture or environment. The spread of the novel coronavirus disease (COVID19) has been a challenge that requires an emergent deployment of diagnostic and therapeutic options available. Development of a simple and sensitive immunodiagnostic method based on nanozymes can be useful for monitoring COVID19. Recently, a nanozyme chemiluminescence immunosensor for rapid and portable detection of SARS-CoV-2 spike antigen (S antigen) is developed [109]. The test paper platform based on a peroxidase nanozyme combines traditional enzymatic chemiluminescence analysis (CLIA) with lateral flow assay, which, facilitates early screening of SARS-CoV-2 infections. Furthermore, nanozymes possess the antiviral activity through catalyzing lipid peroxidation of the viral lipid envelope. Thus, nanozymes have the ability to prevent COVID19 transmission and infection.

## Data Availability

All data generated or analyzed during this study are included in the article.
